# Z-DNA-forming sites identified by ChIP-Seq are associated with actively transcribed regions in the human genome

**DOI:** 10.1093/dnares/dsw031

**Published:** 2016-07-03

**Authors:** So-I. Shin, Seokjin Ham, Jihwan Park, Seong Hye Seo, Chae Hyun Lim, Hyeongrin Jeon, Jounghyun Huh, Tae-Young Roh

**Affiliations:** ^1^Department of Life Sciences; ^2^Division of Integrative Biosciences & Biotechnology, Pohang University of Science and Technology (POSTECH), Pohang, Republic of Korea

**Keywords:** Z-DNA, Z-DNA-binding protein, human genome, ChIP-Seq, active transcription

## Abstract

Z-DNA, a left-handed double helical DNA is structurally different from the most abundant B-DNA. Z-DNA has been known to play a significant role in transcription and genome stability but the biological meaning and positions of Z-DNA-forming sites (ZFSs) in the human genome has not been fully explored. To obtain genome-wide map of ZFSs, Zaa with two Z-DNA-binding domains was used for ChIP-Seq analysis. A total of 391 ZFSs were found and their functions were examined *in vivo*. A large portion of ZFSs was enriched in the promoter regions and contain sequences with high potential to form Z-DNA. Genes containing ZFSs were occupied by RNA polymerase II at the promoters and showed high levels of expression. Moreover, ZFSs were significantly related to active histone marks such as H3K4me3 and H3K9ac. The association of Z-DNA with active transcription was confirmed by the reporter assay system. Overall, our results suggest that Z-DNA formation depends on chromatin structure as well as sequence composition, and is associated with active transcription in human cells. The global information about ZFSs positioning will provide a useful resource for further understanding of DNA structure-dependent transcriptional regulation.

## 1. Introduction

Z-DNA is a structural form of DNA that has a left-handed double helix and a zigzagging sugar-phosphate backbone. The bases in Z-DNA alternate between an *anti* and *syn* orientation about the N-glycosidic bond, whereas in B-DNA, they uniformly have an *anti*-conformation. Z-DNA easily occurs in alternating purine-pyrimidine sequences, with those sequences’ propensity for forming Z-DNA in the following order: d(GC)n > d(CA)n > d(CGGG)n > d(AT)n.^1–3^ Z-DNA formation requires more energy than B-DNA formation, but Z-DNA can be stable under particular conditions, including those with high salt concentrations, divalent and polyvalent ions, dehydrating agents, and DNA-specific conditions such as the presence of chemically modified bases and negative supercoiling.^4^^,^^5^

A family of proteins has the ability to bind Z-DNA through a Z-DNA-binding domain. One such protein is the human double-stranded RNA adenosine deaminase (hADAR1), which catalyzes the deamination of adenosine to inosine in double-stranded RNA. The N-terminal region of this enzyme has a Z-DNA-binding domain composed of Zα and Zβ motifs. Zα belongs to the winged helix-turn-helix family of proteins and recognizes *syn*-conformed purine residues, meaning it binds specifically to Z-DNA, whereas Zβ does not.^6^ Za (residues 133–209) binds to Z-DNA better than Zα (residues 121–197). Zaa can be generated by replacing Zβ with Zα, resulting in a much higher affinity and specificity to Z-DNA than Zα.^7^^,^^8^ Za and Zaa have been widely used as probes for Z structure *in vitro* and *in vivo*. Motifs similar to the Zα of hADAR1 are also present in other Z-DNA-binding proteins, such as E3L and DLM1, and are important for the proteins’ biological functions.^9^^,^^10^ Because Za of hADAR1 has a strong affinity to Z-DNA and its characteristics have been extensively studied, Za is useful for identifying Z-DNA-forming sites in cells.

In human cells, Z-DNA-forming regions (ZDRs) have been predicted genome-wide by the computer programs Z-hunt and Z-catcher. Potential ZDRs are enriched near the transcriptional start sites (TSSs) of genes.^11^^,^^12^ This suggests that Z-DNA functions in transcriptional regulation, and indeed, RNA polymerase II accumulates local negative supercoiling behind it as it moves along the DNA strand during transcription, creating a favourable environment for Z-DNA formation.^13^ In addition, experiments show that Z-DNA formation, as detected by a Z-DNA antibody, increases with the expression level of the *c-myc* gene,^14^ and Z-DNA formation, enhanced by the BAF complex, plays a role in stabilizing open chromatin, giving RNA polymerase II access to the *CSF1* gene.^15^ In a similar manner, transcription of the *HO-1* gene is increased by Z-DNA formation.^16^ Furthermore, the incorporation of Z-DNA into nucleosomes is unfavourable, resulting in the promoter region Z-DNA regulating transcription by establishing the boundaries of neighboring nucleosomes and producing an open chromatin state.^17^ In addition, Z-DNA is involved in stimulating homologous recombination, protecting the genome, and genetic instability.^18–20^

Recently, 186 Z-DNA hotspots in human cells were identified using Zα_ADAR1_ as a probe in an *in vitro* chromatin affinity precipitation (ChAP)-Sanger sequencing experiment.^12^ Among them, 46 hotspots were located in the centromere and were correlated with high densities of single nucleotide polymorphisms (SNPs), a finding that was inconsistent with the *in silico* predictions of ZDRs being located mostly in TSSs.

Because ZFSs have never been explored at the human genome level by high-throughput analysis, it is difficult to completely understand the biological functions of Z-DNA. Sanger sequencing technique is low throughput, making the interpretation of the sparse data difficult. In order to overcome this limitation, we used chromatin immunoprecipitation with Zaa that consists of two copies of Za, followed by next-generation sequencing (ChIP-Seq). This method provided information on ZFSs in the human genome at high resolution and coverage. We found ZFSs with high confidence, and determined their genomic and epigenetic features. Our results support the positive correlation between Z-DNA formation and active transcription in human cells.

## 2. Materials and methods

### 2.1. Cell culture and the expression of Zaa

HeLa cells, a human cervix carcinoma cell line, were cultured at 37 °C with 5% CO_2_ in DMEM media containing 10% FBS, 50 U/ml penicillin, and 50 μg/ml streptomycin. For transfection, Zaa were amplified by PCR using Zaa-Fok as a template, generated by Mulholland *et al.*^21^ PCR products were digested with BclI and XbaI, and cloned into the BamHI/XbaI site of pEF1α, in which a FLAG tag was added. The SV40 nuclear localization signal (NLS) sequence was generated as described^16^ and inserted into the XmaI/AscI site of pEF1α, producing pEF1α-Zaa. pEF1α-Zaa was transfected into HeLa cells using GeneJuice (Novagen, Germany).

### *2.2. In vitro* Z-DNA cleavage assay

pET28a-Fok was constructed by inserting a catalytic domain of *Fok*I endonuclase into the HindIII/XhoI site of pET28a and a (Gly_4_Ser)_3_ linker was added upstream of Fok by cloning into the EcoRI/HindIII site, as described in Mulholland *et al*.^21^ and Kim *et al.*^22^ For generation of pET28aZa-Fok and pET28aZaa-Fok, Za or Zaa was placed in front of the (Gly_4_Ser)_3_ linker by inserting Za or Zaa into the NheI/EcoRI site in pET28a-Fok. BL21 (DE3) bacterial cells containing pET28a-Fok, pET28a-Za-Fok, or pET28a-Zaa-Fok were grown at 37 °C and induced with 0.1 mM IPTG and grown for 12 hr at 18 °C. Fok, Za-Fok, and Zaa-Fok were purified using His-Bind resin (Novagen) charged with Ni^2+^. Purified recombinant proteins were dialyzed and analyzed using SDS-polyacrylamide gel electrophoresis followed by Coomassie Blue staining. Aliquots were stored at -80 °C. pDHg16 and pDPL6 were obtained from Addgene (USA). The supercoiled form of pDHg16 was purified using the LaboPass Mini Kit (Cosmo Genetech, Korea). The linear form was prepared by digesting supercoiled pDHg16 with PstI. For the *in vitro* Z-DNA cleavage assay, supercoiled or linear plasmid was incubated with Fok, Za-Fok, or Zaa-Fok in digestion buffer (10 mM Tris-Cl [pH 8.0], 50 mM KCl, 1 mM DTT, 2.5% glycerol and 0.05% NP40) at 22 °C. After 20 min, MgCl_2_ was added to a final concentration of 10 mM, and the reaction was further incubated for 2 hr. Fok, Za-Fok, or Zaa-Fok was inactivated by heat treatment at 50 °C for 30 min. The supercoiled plasmid DNA was digested with PstI for 1 hr at 37 °C and analyzed by gel electrophoresis in 1% agarose gel. For a generation of pDPL6-ZFSs and pDPL6-negative, predicted short ZDR sequences inside ZFSs or a sequence without potential to form Z-DNA was inserted into the XbaI/SalI site of pDPL6. The resulting pDPL6-ZDRs and pDPL6-negative were used for *in vitro* Z-DNA cleavage assay.

### 2.3. Immunofluorescence analysis and chromatin immunoprecipitation (ChIP)

The expression of Zaa in HeLa cells was monitored by immunofluorescence analysis. Forty hours after transfection, HeLa cells were fixed in 4% paraformaldehyde for 10 min at room temperature and then permeabilized with 0.1% Triton X-100 for another 10 min at room temperature. Cells were incubated with blocking solution (0.1% BSA in PBS) for 1 hr at room temperature and sequentially incubated with FLAG M2 antibody in blocking buffer for 2 hr at 37 °C, followed by incubation with Dylight 488–labelled secondary antibody (Abcam, UK) for 1 hr at 37 °C. Nuclei were stained with Hoechst dye, and samples were observed using the Olympus FluoView 1200 confocal microscope. ChIP was performed as described^23^ with small changes. Briefly, transfected cells were cross-linked with 1% formaldehyde for 10 min at room temperature. Cell fixation was stopped by adding 2.5 M glycine to a final concentration of 0.1375 M and incubating for 5 min. After, cells were washed twice with cold PBS and collected. The cell nuclei were extracted with buffer 1 (10 mM HEPES [pH 6.5], 0.25% Triton X-100, 10 mM EDTA, 0.5 mM EGTA, and 1 mM PMSF) and buffer 2 (10 mM HEPES [pH 6.5], 200 mM NaCl, 1 mM EDTA, 0.5 mM EGTA, and 1 mM PMSF), and isolated nuclei pellets were resuspended in sonication buffer (50 mM HEPES [pH 7.9], 140 mM NaCl, 1 mM EDTA, 1% Triton X-100, 0.1% Na-deoxycholate, 0.1% SDS, and 1× protease inhibitor cocktail). Chromatin with a range from 100 to 300 bp was prepared by sonication and subjected to immunoprecipitation with 2 μg of FLAG M2 (Sigma, USA), normal mouse IgG (Santa Cruz, USA), or RNA polymerase II (8WG16, Millipore, USA) antibodies and protein A/G magnetic beads (Thermo, USA). One percent of sonicated chromatin was reserved as input before immunoprecipitation. Proteinase K was added to ChIP and input samples, and the chromatin was reverse-crosslinked by incubating for 10 hr at 65 °C in buffer 4 (10 mM Tris-Cl [8.0], 1 mM EDTA, 0.5% SDS). ChIPed DNA and input DNA was purified and used for PCR or sequencing library construction. The primers for Zaa ChIP-qPCR were shown in Supplementary Table S1.

### 2.4. ChIP-Seq

The sequencing library was prepared as described^24^ with a few modifications. ChIPed DNA and input DNA fragments were end-repaired, dA tailed, and ligated with the genomic DNA adapters provided by Illumina (USA). Adapter-ligated DNA fragments were purified and amplified by PCR. Gel extraction was performed to isolate 200–400-bp PCR products. Libraries were sequenced using the Illumina Genome Analyzer IIx and HiSeq 2500. Sequence alignment was performed using Bowtie2 (v2.25) on the human genome (hg18). The sequencing data of Zaa rep1, rep2, rep3, input and IgG were deposited in the NCBI GEO database (GSE71682). The overall scheme of ChIP-Seq is shown in Fig. 2C.

### 2.5. Data analysis

The ChIP-Seq data was summarized in Supplementary Table S2. Uniquely mappable reads were used for the following computational analyses. Using IgG data as a control, Zaa peaks on each Zaa replicate were detected by SPP (v.1.10.1) with the following options: -x:-500:85, -npeak = 300,000. Input DNA was also used as a control but IgG data was applied to further analysis for practical comparison purpose.^25^ Reproducible Zaa peaks between replicates were determined by Irreproducible Discovery Rate (IDR) analysis.^26^ Self-consistent peaks between two pseudoreplicates (half of mapped reads in each replicate) were found using IDR ≤ 0.01. Peaks consistent between pooled-pseudoreplicates (half of mapped reads pooled across all replicates) were found using IDR ≤ 0.001. In addition, peaks consistent between three pairs of Zaa replicates were found using IDR ≤ 0.05. Of three consistent peak numbers, the maximum peak number was used as a cutoff. With this cutoff, top-ranked peaks were selected in the pooled replicates. Then, the Zaa peaks were examined if they were overlapped with frequently overrepresented random peaks called blacklisted regions. The blacklist detected in the human genome was downloaded from the website (https://sites.google.com/site/anshulkundaje/projects/blacklists, 26 April 2016, date last accessed). After filtering, the final 391 Zaa peaks were selected as ZFSs and used for further analysis. The Pearson correlation coefficient was calculated by counting reads mapped within ZFSs for every sample and comparing those read counts across samples.

Genes containing ZFSs near the transcription start site (± 2 kb) were subjected to a Gene Ontology enrichment analysis by DAVID. Known motifs that were significantly enriched in ZFSs were examined by Homer (v4.8). The presence of ZDRs within the ZFSs were predicted by Z-catcher with two cutoff values for the negative supercoiling density, -0.08 or -0.07. For comparison, 10,000 random peaks were selected arbitrarily with the distribution of length of ZFSs. The supercoiling density value required for Z-DNA formation was calculated for ZFSs and random peak regions. The region 2 kb upstream and downstream from the centre of each ZFS and random peak was divided into 20-bp windows. The supercoiling density value was calculated in a 20-bp sliding window overlapped with 18-bp. The value for each window was plotted.

For the gene expression analysis, public mRNA-Seq data was downloaded and analyzed.^27^ Sequence reads were mapped by TopHat v2.0.9 program ^28^ to the human genome reference sequence (hg18). Quantification and normalization for each transcript were performed using the RPKM method (Reads Per Kilobase of exon per Million aligned sequence tags). The colocalization of ZFSs with RNA polymerase II binding sites and histone modifications was examined using the following ChIP-Seq datasets; RNA Polymerase II (SRR349806, SRR349811, and SRR765748) and histones H3K4me3, H3K9ac, H3K9me3, H3K27me3, H3K36me3, and H3K79me3 (SRP002252). The heatmap was drawn by calculating the normalized read counts for 50-bp windows within ZFSs and 3 kb upstream and downstream.

### 2.6. Luciferase reporter assay

For the *in vitro* Z-DNA cleavage assay, predicted short ZDRs inside ZFSs and one negative control were selected and cloned into the NheI/XhoI site of pGL3-HS containing minimal heat-shock promoter.^29^ The resulting pGL3-HS-ZFSs or pGL3-HS-negative were transfected into HeLa cells and luciferase reporter assay was performed using Dual-Luciferase Reporter Assay System (Promega, USA). Luciferase activities of each pGL3-HS-ZFSs and pGL3-HS-negative were normalized to those of pGL3-HS and fold change of relative luciferase activity was calculated by dividing the normalized luciferase activity in pGL3-HS-ZFSs by the normalized luciferase activity of pGL3-HS-negative.

## 3. Results and discussion

### 3.1. Recognition of Z-DNA structure by Za and Zaa

**Figure 1:**
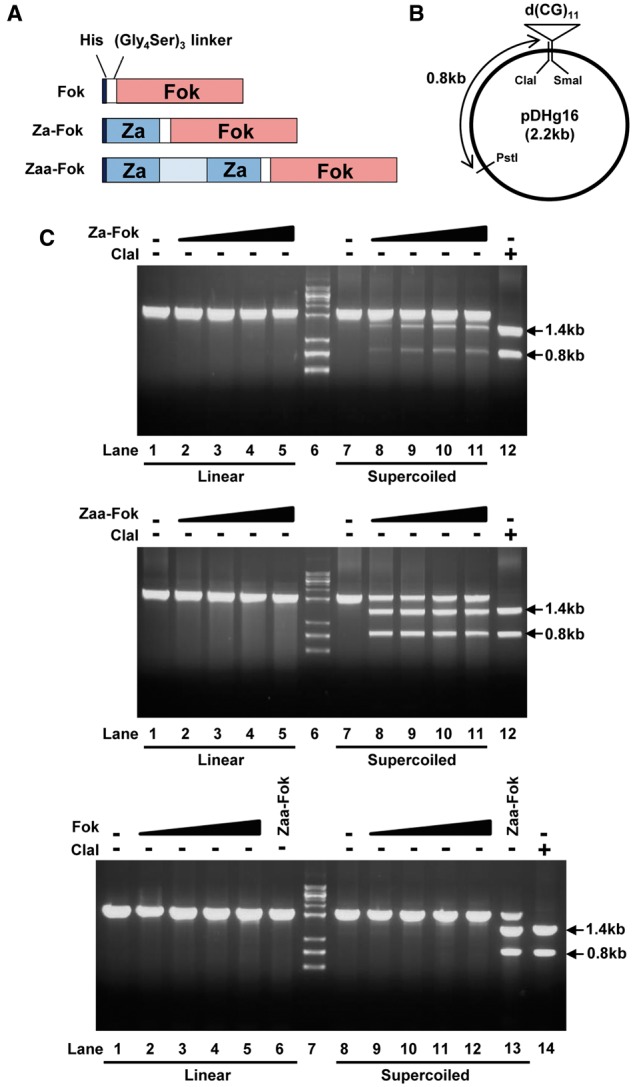
Identification of Z-DNA structure by cleavage with Za- and Zaa-Fok. (A) Schematic constructs of recombinant Za-Fok, Zaa-Fok, and Fok with a His tag and (Gly_4_Ser)_3_ linker. (B) pDHg16 contains CpG dinucleotide repeats. Arrows indicate ClaI and PstI cleavage sites. (C) The *in vitro* Z-DNA cleavage assay was analyzed on a 1% agarose gel. Linear and supercoiled pDHg16 was digested by various concentrations of Za-Fok, Zaa-Fok, or Fok. The molar ratio of Za-Fok, Zaa-Fok, or Fok to pDHg16 was 0, 2, 4, 6 and 8:1 (top and middle, lane 1-5, 7-11, bottom, lane 1-5, 8-12). Arrows indicate the resulting fragments produced by Za-Fok or Zaa-Fok, and PstI digestion (top and bottom, lane 1-5, 7-11). For a positive control, Zaa-Fok was used at the same time that Fok was tested (bottom, lane 6 and 13). Lane 6 (top and middle) and 7 (bottom) has DNA molecular mass markers. Lane 12 (top and middle) and 14 (bottom) has the restriction fragments of ClaI and PstI.

Z-DNA formation and Z-DNA recognition by Za and Zaa were examined using an *in vitro* cleavage assay. The Z-DNA-binding domains Za and Zaa were fused with the catalytic domain of *Fok*I, which is a type IIS restriction endonuclease (Fig. 1A). Two fusion proteins, Za-Fok and Zaa-Fok, and Fok as a negative control were expressed and purified. A d(CG)_11_ insert within pDHg16 is known to form Z-DNA when the plasmid is negatively supercoiled (Fig. 1B). The *in vitro* Z-DNA cleavage assay showed that Za-Fok and Zaa-Fok did not digest the linear form of pDHg16 (Fig. 1C, top and middle, lane 1–5), whereas they cleaved the d(CG)_11_ insert in the negatively supercoiled pDHg16, producing 1.4-kb and 0.8-kb DNA fragments (Fig. 1C, top and middle, lane 7–11). These results suggest that the binding of Za and Zaa is dependent on DNA conformation rather than sequence composition, which is consistent with previously reported data.^7^^,^^8^ As the concentration of Za-Fok was increased, the two bands produced by Za-Fok and PstI became thicker, but the increase in activity stopped when eight times more Za-Fok than pDHg16 was added (Fig. 1C, top, lane 10). Because Zaa has a higher affinity to Z-DNA than Za, the two fragments produced by Zaa-Fok and PstI formed clearer, thicker bands, and Zaa-Fok had more activity than Za-Fok, even at a 2:1 molar ratio of Zaa-Fok:pDHg16 (Fig. 1C, middle, lane 8). Contrary to Za- and Zaa-Fok, digested fragments of supercoiled pDHg16 were not shown with Fok (Fig. 1C, bottom, lane 8-12). Therefore, we confirmed that Za and Zaa were capable of recognizing and binding Z-DNA and that Zaa showed higher affinity to Z-DNA than Za.

### 3.2. Identification of ZFSs in human genome

**Figure 2:**
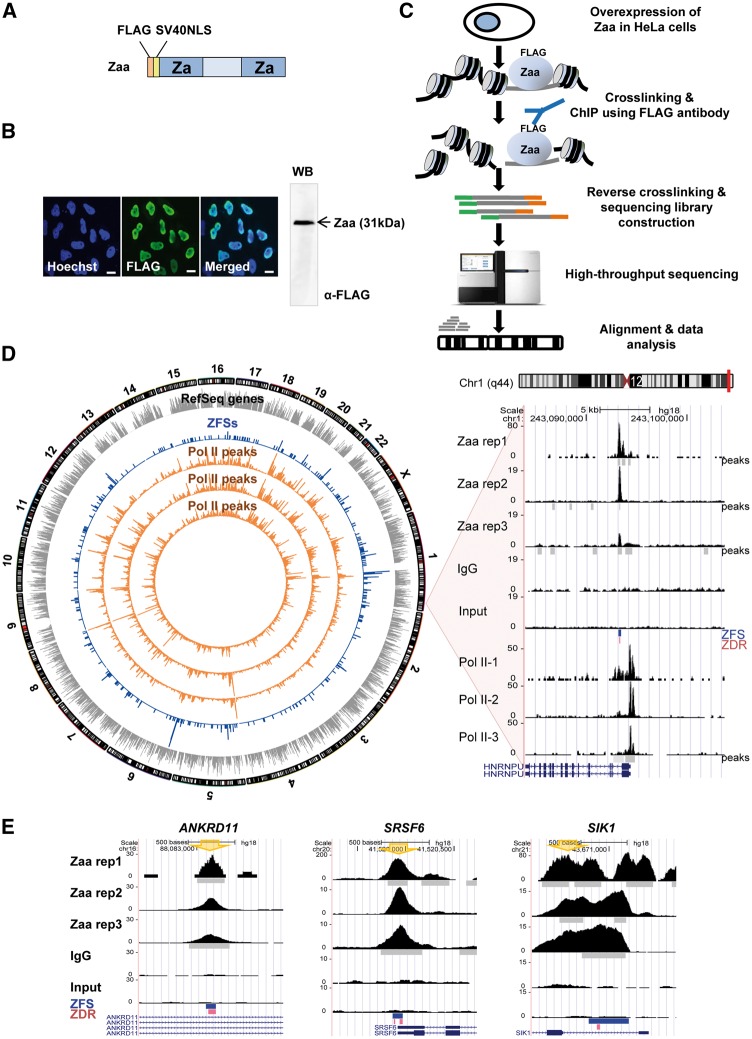
Genome-wide mapping of Z-DNA using Zaa. (A) Schematic of Zaa with FLAG tag and SV40NLS. (B) The localization of Zaa in HeLa cells was observed by confocal microscopy. Zaa was labeled with FLAG antibody, followed by a secondary antibody conjugated to green-fluorescent dye. Nuclei were stained using Hoechst dye (blue). Pictures are representative of three independent experiments. Scale bars indicate 10 μm. The Western blot image is shown on the right panel. (C) Flowchart illustrating the ChIP-Seq process. Zaa construct was transiently transfected into HeLa cells. (D) Circos plot displaying the genome-wide distribution of genes, ZFSs, and polymerase II (gray bar: RefSeq gene, blue bar: ZFSs, orange bar: RNA polymerase II peak). One representative region in chromosome 1 is shown on the UCSC genome browser. The y-axis of the UCSC genome browser is the normalized read count. Peak regions, ZFS, and predicted ZDRs in ZFSs (cutoff of − 0.08) are indicated as gray bars, blue bars, and pink bars, respectively. (E) Examples of ZFSs are shown. The UCSC genome browser was used to visualize read distributions of three Zaa replicates, IgG, and input in *ANKRD11, SRSF6*, and *SIK1*. The PCR primer positions for ChIP validation are marked by big arrow.

The specificity of selectivity of antibody was examined. Zaa was overexpressed and localized mostly in the nucleus in HeLa cells. Confocal microscopic analysis determined that the expressed Zaa accumulated in the nuclei, confirming a high level of expression and nuclear localization of Zaa. Additionally, non-specific protein bands were not observed using FLAG antibody, indicating that the antibody is highly specific to Zaa (Fig. 2A and B). Even though some commercial antibodies against Z-DNA are available and several groups have succeeded in detecting Z-DNA in human cells,^14^^,^^30^ it is hard to identify sites enriched for Z-DNA-antibody binding in HeLa cells (data not shown), likely because the intensive sonication required for fragmenting chromatin may cause Z-DNA more unstable. Therefore, we used Z-DNA-binding proteins as probes for detecting Z-DNA-forming sites. ChIP-Seq experiments were performed with Zaa-transfected HeLa cells (Fig. 2C) and sequencing data were obtained from three biological replicates and two negative controls, IgG and Input DNA. To identify confident ZFSs, IDR framework analysis was applied to find Zaa peaks consistent between replicates. The numbers of self-consistent peaks between two pseudoreplicates were 1,024 for Zaa repl1, 520 for Zaa rep2, and 814 for Zaa rep3 (IDR ≤ 0.01) and the number of peaks consistent between pooled-pseudoreplicates is 649 (IDR ≤ 0.001). Additionally, the numbers of peaks consistent between pairs of Zaa replicates are 197 between rep1 and rep2, 229 between rep1 and rep3, and 427 between rep2 and rep3 (IDR ≤ 0.05). Of these three, the maximum peak number (427) was used as a cutoff value. Top 427 Zaa peaks in pooled Zaa replicates were further processed with blacklist regions and finally 391 regions were selected as ZFSs (Supplementary Fig. S1). The list of 391 ZFSs and their surrounding genomic features is shown in Supplementary Table S3. We confirmed that the correlations of mapped reads were higher between Zaa replicate pairs than between unrelated sample pairs (Supplementary Fig. S2). The Pearson’s correlation coefficients between Zaa replicates were shown; 0.962 for rep1-rep2, 0.774 for rep1-rep3, 0.814 for rep2-rep3, 0.375 for rep1-IgG, 0.379 for rep2-IgG, and 0.342 for rep3-IgG, 0.385 for rep1-Input, 0.421 for rep2-Input, 0.295 for rep3-Input, and 0.760 for IgG-Input. The correlation coefficients are much higher between Zaa replicates than among pairs of replicate-control. As shown in the comprehensive genome-wide view of ZFSs, the overall ZFSs were distributed all over the chromosome (Fig. 2D). As examples, sequence tag distributions of replicates, input and IgG are visualized by the UCSC genome browser (Fig. 2E, Supplementary Fig. S3).

### 3.3. Validation of ZFSs by Zaa ChIP-qPCR and an *in vitro* Z-DNA cleavage assay

In order to validate ZFSs, the existence of predicted ZDRs in ZFSs was first examined using the Z-catcher program. The advantage of Z-catcher is that it can predict ZDRs genome-wide using different σ levels which specify the superhelical density, a proxy for the energy that can cause a B-to-Z transition. About 80% and 50% of ZFSs contained more than one predicted ZDR at the cutoff of σ = −0.08 and −0.07, respectively. d(CG) or d(CA/TG) repeating sequence is well known to form Z-DNA. However, little is known about the Z-DNA formation in other sequences, except for alternating repeat sequences. To compare the efficiency of Z-DNA formation between different sequences, we randomly selected 10 ZFSs for validation and then divided them into two groups based on the number of alternating purine/pyrimidine dinucleotides of predicted ZDRs: an alternating (Pu)(Py) group (≥ 6 dinucleotides) and a non-alternating (Pu)(Py) group (< 6 dinucleotides). Supplementary Table S4 lists the genes that were closest to each ZFS and the predicted ZDR sequence. Four negative controls were designed not to contain any Zaa peaks, 5–10 kb away from a ZFS. ChIP-qPCR found that Zaa was significantly enriched in 9 out of 10 ZFSs (90%), relative to the average of the ZFS-negative regions (*P*-value < 0.05) (Supplementary Fig. S4A, marked by cross). The control IgG had low Zaa enrichment for all ZFSs. There was little difference between the enrichment levels of Zaa in the alternating (Pu)(Py) group and the non-alternating (Pu)(Py) group, even though the predicted ZDR sequences were not identical and could not be directly compared. Among nine Zaa-enriched ZFSs (*ANKRD11*, *ROR*, *SNX12, SRSF6*, TFAP2A, *SIK1*, *HIST2H2AC*, *STX16*, *and PIM3*), the most enriched ZFSs by Zaa is *SIK1* whose ZDR does not contain alternating purine/pyrimidine repeat sequences. In addition, *STX16* and *PIM3* also show relatively high levels of Zaa enrichment. The enrichment level of Zaa seemed not to be closely correlated with the presence of an alternating purine/pyrimidine repeat sequence. Presumably, alternating purine/pyrimidine repeat sequences might not be the only major factor required to form the Z-DNA structure in cells.

We next performed an *in vitro* Z-DNA cleavage assay to confirm whether the predicted ZDRs in ZFSs were able to form Z-DNA in a negatively supercoiled plasmid. Zaa-Fok digested 4 of the 5 predicted ZDRs in the alternating (Pu)(Py) group (pDPL6-*ANKRD11*, -*ROR1*, -*SNX12*, and -*SRSF6*; each ZDR labeled by its corresponding gene name) and 2 of the 5 predicted ZDRs in the non-alternating (Pu)(Py) group (pDPL6-*SIK1* and -*PIM3*) (Supplementary Fig. S4B). In contrast to the supercoiled forms of the pDPL6-ZDRs, the supercoiled pDPL6-negative was not digested by Zaa-Fok. Therefore, 6 out of the 10 predicted ZDRs (60%) formed the Z-DNA structure in negatively supercoiled plasmids. The intensities of the two bands produced by Zaa-Fok digestion were quantitated (Supplementary Fig. S4C). The digestion efficiency of Zaa-Fok for each predicted ZDR had a low correlation with the enrichment level of Zaa (Supplementary Fig. S4D). For example, pDPL6-*SIK1* was digested by Zaa-Fok with a similar digestion efficiency as pDPL6-*SRSF6*, meaning that they had sequences with similar potential to form Z-DNA *in vitro*. However, the ZFS in *SIK1* had more Zaa enrichment than the ZFSs in *SRSF6*, as demonstrated by ChIP-qPCR. This difference between the *in vitro* Z-DNA cleavage assay and the Zaa ChIP-qPCR might be caused by the ZFSs located in the chromatin context in cells. Contrary to Zaa ChIP-qPCR, only 60% of predicted ZDRs in the ZFSs were confirmed by an *in vitro* Z-DNA cleavage assay. The remaining 40% of predicted ZDRs were not digested by Zaa-Fok probably because the ZDR sequences *per se* had a low potential to form Z-DNA and levels of negative supercoiling were restricted *in vitro*. However, the formation of DNA supercoiling *in vivo* is affected by chromatin structure related with biological processes so that supercoil density might dynamically change. In the presence of a high degree of negative supercoiling, Z-DNA can be formed even at ZDRs with the relatively low potential to form Z-DNA.

Consequently, Z-DNA formation in cells may be affected by the dynamic regulation of chromatin structure, such as chromatin remodeling and nucleosome positioning, in addition to the sequence composition and length of the ZDRs. In summary, we confirmed that the enrichment level of Zaa was not simply determined by the existence of known alternating purine/pyrimidine repeat sequences in ZFSs.

### 3.4. Characterization of genomic features of ZFSs 

**Figure 3:**
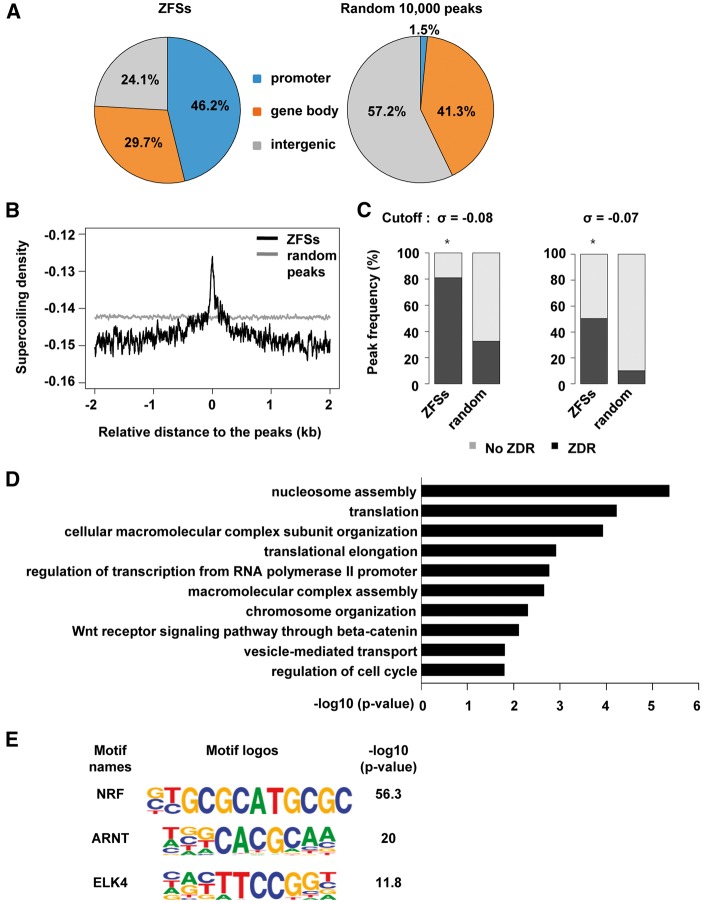
Genomic features at ZFSs. (A) The pie charts show the genomic distribution of ZFSs and 10,000 random peaks. About 46% of ZFSs were found in promoter regions which are defined as within 2 kb of the TSS. (B) The supercoiling density was analyzed as a function of the distance from the peak using Z-catcher. The centre of the peaks is at zero. (C) Peak frequency indicates the number of ZFSs or random peaks that contain predicted ZDRs (black bar). Predicted ZDRs in peak regions were identified using the cutoff of -0.07 or -0.08 in Z-catcher. At -0.08, ZFSs and random peaks containing predicted ZDRs accounted for 81.07% and 32.58% of peaks, respectively. At -0.07, ZFSs and random peaks containing predicted ZDRs accounted for 50.38% and 10.09% of peaks, respectively. A two-sample proportion test was performed using a 99% confidence level. Stars indicate Fisher’s *P*-value < 2.2e-16. (D) Gene ontology (GO) analysis was performed using the DAVID functional annotation tool with genes that had a ZFS within 2 kb of their TSS. The x-axis indicates −log10 *P*-value and the y-axis displays the enriched GO terms. (E) Known motif enrichment analysis was carried out using Homer. The top three motifs for transcription factors in ZFSs are shown. Enrichment is shown as −log10 *P*-value. Letter size indicates the frequency of nucleotides.

ZFSs were significantly enriched in the promoter regions of genes (46.2%), in the gene body (29.7%), and in intergenic regions (24.1%). On the other hand, only 1.5% of random peaks were present in promoter regions, and a large portion of random peaks were found in intergenic regions (57.2%; Fig. 3A). As demonstrated by the *in vitro* Z-DNA cleavage assay (Fig. 1C and Supplementary Fig. S4B), negative supercoiling is required to induce Z-DNA formation. Thus, ZFSs are likely to be negatively supercoiled. We analyzed the distribution of the superhelical density in and around ZFSs as well as random peaks. Because, in Z-catcher, the free energy requirement for B-to-Z transition was translated into the superhelical density (σ) which provides energy for the transition, given sequences which require less supercoiling (closer to zero) have greater Z-DNA forming potentials. As shown in Fig. 3B, the superhelical density required for Z-DNA formation was lower in the centre of ZFSs than random peaks, meaning that ZFSs have concentrations of Z-DNA forming potentials. In addition, the existence of short ZDRs in 391 ZFSs was examined using the Z-catcher. Among ZFSs, 81.0% carried potential ZDRs with the cutoff of −0.08, but only 32.5% of random peaks had potential ZDRs. When the cutoff value was increased to −0.07, 50.3% of ZFSs and 10.0% of random peaks contain predicted ZDRs. However, considering the ratio of ZFS to random peak ratio in frequency at the cut off of −0.07, 5 times more ZFSs had ZDRs than random peaks (Chi-Square *P*-value: 1.60e-131) whereas ZFSs containing predicted ZDRs are just 2.5 times more than random peaks at the cut off of -0.08 (Chi-Square *P*-value: 2.88e-87, Fig. 3C). Thus, ZFSs significantly contain sequences with higher potential for forming Z-DNA than random peaks.

A functional association of ZFSs with gene was examined by GO analysis. As Fig. 3D is shown, Z-DNA may be involved in a wide variety of biological processes such as nucleosome assembly, translation, cellular macromolecular complex subunit organization, translational elongation, and the regulation of transcription from RNA polymerase II promoters, *etc*. Some studies have reported that transcription factors and remodeling complexes need Z-DNA-forming sequences to bind DNA.^15^^,^^16^ Based on this, we hypothesized that known and unknown factors are binding within ZFSs. We analyzed the ZFSs for transcription factor binding motifs. As shown in Fig. 3E, the NRF motif is the most enriched motif in ZFSs. In addition, there are other transcription factor binding motifs for ARNT and ELK4. These proteins are interesting candidates for follow-up studies on the association of Z-DNA to transcription. According to the result of *de novo* motif analysis, about 40% of ZFSs contained alternating purine/pyrimidine repeat sequences which were not found in the remaining ZFSs (Supplementary Fig. S5). This result is also consistent with the Zaa ChIP-qPCR assay; alternating purine/pyrimidine repeat sequences might not be the only major factor required to form the Z-DNA structure in cells.

Negative supercoils are generated behind RNA polymerase II as it moves along the DNA strand and during the nucleosome remodelling.^31^^,^^32^ Furthermore, negative supercoiling is found in open chromatin regions, which contain actively transcribed genes.^33^^,^^34^ Z-DNA is known to be stabilized by negative supercoiling, as determined *in vivo*. Our experiments demonstrating that Zaa-Fok digests predicted ZDRs in a negatively supercoiled plasmid and that ZFSs have high potential to form Z-DNA also support the assertion that Z-DNA can form during negative supercoiling. Moreover, we found a large portion of ZFSs were located in promoter regions. All of these results suggest the possibility that the Z-DNA formation is related to active transcription.

Recently, it has been reported that false-positive signals from ChIP-seq could be caused by artefacts and biases inherent in the ChIP-seq procedures such as batch-to-batch variation in sonication efficiency, cross-reactivity of antibodies with non-specific proteins, differential chromatin accessibility, and so on.^35^^,^^36^ Even though input and IgG controls are used, artificially high ChIP signals cannot be fully removed. However, most Zaa peaks (91%) were not overlapped with ultra-high signal artefact regions (data not shown), which suggests that Zaa peaks are reliable to find a biological function of Z-DNA.

### 3.5. Differences of distribution of ZFSs and Z-DNA hotspots

Z-DNA hotspots found by ChAP and cloning, followed by Sanger sequencing, has been reported that they were mainly found in centromeres and telomeres.^12^ Because many of our ZFSs were detected in the promoter regions of human genes and Zaa enrichment in several Z-DNA hotspots was not observed in our Zaa ChIP-qPCR data, we further examined this discrepancy. Out of 186 Z-DNA hotspots, only 6 Zaa peaks were colocalized with Z-DNA hotspots (Supplementary Fig. S6A). Notably, our input and IgG also showed strong read enrichment in Z-DNA hotspots (Supplementary Fig. S6B).

There are four possible implications of such differences; First, different methods were used for detecting Z-DNA in human cells. Li *et al.* used dual cross-linking ChAP followed by cloning and Sanger sequencing. Because this method has less coverage than ChIP-Seq, it is possible that Za-binding regions were only locally mapped, which might not give enough information for identifying the role of Z-DNA. Second, they applied purified Za to fixed and permeabilized cells and then cross-linked Za to DNA. This strategy might result in identifying Z-DNA formed in other genomic locations than the locations found in our approach. Third, different constructs were used to detect Z-DNA. Since Zaa more strongly and specifically binds to Z-DNA than Za, different maps of Z-DNA forming sites might be generated by Za and Zaa. Finally, different cell lines were used. Different cells have different characteristics, and Z-DNA locations may be associated with different biological processes. Future studies are needed to find Z-DNA-forming sites and to compare the biological functions of Z-DNA in different cells using each method.

### 3.6. Association of ZFSs with active transcription

**Figure 4:**
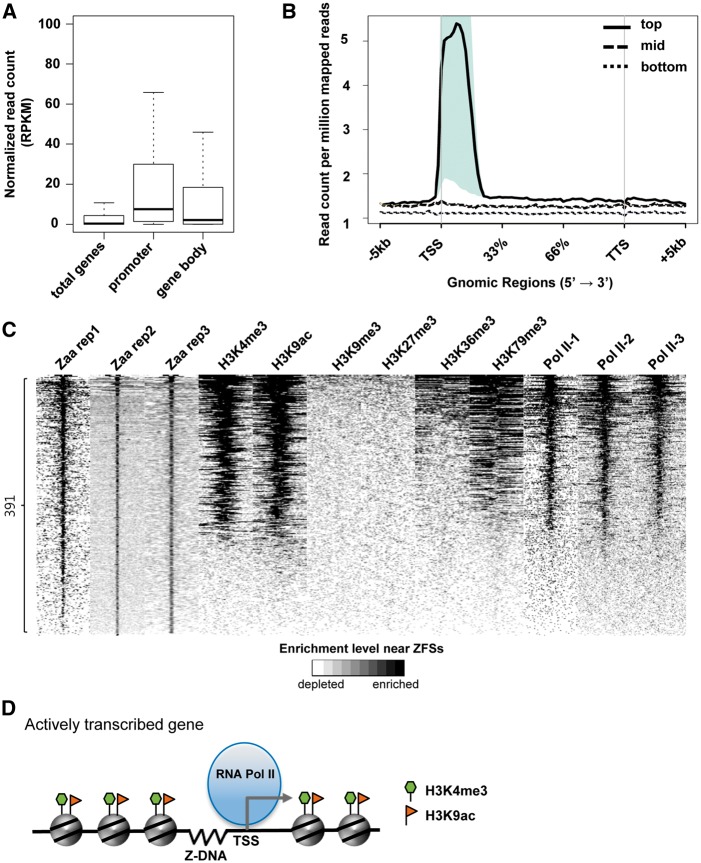
Association of ZFSs with active transcriptional regions. (A) Gene expression analysis was performed. Public mRNA sequencing data were downloaded and mapped to the human reference genome. Each transcript was quantified and normalized using the RPKM (reads per kilobase per million). ‘Promoter’ or ‘Gene body’ indicates where the genes have the ZFS. (B) Genes were ranked based on their RPKM values and divided into three groups based on their expression: top (1,000 highest expressing genes, thick line), mid (1,000 genes between the top and bottom group, dashed line), bottom (6,661 unexpressed genes (RPKM = 0), dotted line). Reads of each Zaa replicate were normalized by Read count per million mapped reads and normalized reads were counted within 5 kb of the TSSs. Shaded regions indicate *P*-value < 0.05. (C) Heatmap showing the enrichment of various histone marks and RNA polymerase II at ZFSs. ZFSs were ranked by the enrichment of Zaa, calculated using normalized read counts in ZFS ± 3kb. The enrichment level is shown by the color scale (white: depletion, black: enrichment). (D) The correlation of Z-DNA with RNA polymerase II and histone marks is illustrated. In promoter regions of actively transcribed genes, Z-DNA is associated with RNA polymerase II and active histone marks such as H3K4me3 and H3K9ac.

To examine the relationship between Z-DNA and transcription, the expression levels of genes associated with ZFSs were analyzed. Higher gene expression was observed in genes with ZFS at their promoter than in the gene body (Mann-Whitney U test *P*-values for promoter vs. gene body, total genes vs. gene body, and total genes vs. promoter are 2.201e-03, 4.322e-06, and < 2.2e-16, respectively. Fig. 4A). Genes were divided into three groups (‘top’, ‘mid’, and ‘bottom’) according to their expression levels and normalized read counts in ZFSs for each Zaa replicate were plotted depending on the gene expression level. The reads mapped in ZFSs were highly enriched in the ‘top’ gene group, especially at the TSSs (Fig. 4B). The same pattern is also shown in other two Zaa replicates (Supplementary Fig. S7). Next, we investigated whether ZFSs are associated with RNA polymerase II deposition. RNA polymerase II ChIP-Seq data generated from three different data sets (Pol II-1, Pol II-2, and Pol II-3) were analyzed. Among the 391 ZFSs, 231 ZFSs (59%) overlapped with the RNA polymerase II peaks (*P*-value < 1.0E-05, Fisher’s exact test, Supplementary Table S5). ZFSs were substantially correlated with RNA polymerase II binding sites, as shown in the Circos diagram and an example region in chromosome 1 (Fig. 2D). Additionally, the association of ZFSs with RNA polymerase II binding sites was experimentally validated in the top seven genes, which had been selected according to Zaa enrichment in the Zaa ChIP-qPCR (Supplementary Fig. S4A). First, as the binding of RNA polymerase II at Zaa peaks was examined in all seven ZFSs via the UCSC genome browser, and the position of RNA polymerase II was overlapped with most ZFSs (Supplementary Fig. S8A). By ChIP-qPCR, RNA polymerase II mostly showed significant enrichment near selected ZFSs (Supplementary Fig. S8B). Therefore, these results confirm that Z-DNA formation is strongly correlated with active transcription.

As the chromatin structure has been suggested to be one of the important factors in Z-DNA formation and our Zaa ChIP-qPCR and the *in vitro* Z-DNA cleavage assay implied the involvement of other factors than nucleotide composition, we tested if specific epigenetic marks were associated with Z-DNA by analyzing histone ChIP-Seq data. Zaa-enriched regions were strongly correlated with the accumulation of H3K4me3 and H3K9ac, which are mainly found in the promoter and enhancer regions of actively transcribed genes. On the other hand, Zaa-enriched regions were not associated with the histone marks H3K9me3, H3K27me3, H3K36me3 and H3K79me3, which are largely in the gene body or are repressive modifications. Moreover, the enrichment of RNA polymerase II was again found in Zaa-enriched regions (Fig. 4C).

The association of Z-DNA formation with active transcription was further examined by luciferase reporter assay. As shown in Supplementary Figure S9, 90% ZFSs (9/10) showed significantly increased luciferase reporter activities relative to the negative control, supporting a positive correlation between Z-DNA formation and transcriptional activation. Collectively, it is evident that the Z-DNA formation is tightly linked with the open chromatin structure of active transcription. Z-DNA is formed near the TSS of actively transcribed genes, with RNA polymerase II and active histone marks located in the promoter regions and, importantly, Z-DNA formation might play a crucial role in activating transcription (Fig. 4D).

### 3.7. Conclusion

Z-DNA is one of the most well studied non-B-DNA structures, and *in vivo* and *in vitro* formation of Z-DNA has been reported in many studies. However, the general biological function of Z-DNA in human cells has not been well elucidated because only a few studies have investigated the genome-wide distribution of Z-DNA-forming sites. Using Zaa as a probe, we identified highly confident 391 ZFSs genome-wide in HeLa cells with ChIP-seq. The majority of 10 ZFSs was validated by Zaa ChIP-qPCR and an *in vitro* Z-DNA cleavage assay. We found that ZFSs are mostly located in promoter regions and contain sequences with the highest potential to form Z-DNA. ZFSs are significantly associated with highly expressed genes and have an enrichment of RNA polymerase II binding. In addition, active histone marks were significantly correlated with Z-DNA formation, meaning that Z-DNA formation in cells relies not only on sequence composition, but also on chromatin structure. Furthermore, we confirmed that the Z-DNA formation might transcriptionally activate genes. These results suggest that there is a strong correlation between Z-DNA formation and active transcription.

This ZFS mapping information could be useful for identifying disease genes whose expression can be altered if Z-DNA formation can be regulated by drugs such as inhibitors of epigenetic regulators, topoisomerase inhibitor, or other reagents that release negative supercoils.

## Conflic of Interest

None declared

## Accession number

The accession number of ChIP-Seq data is GSE71682.

## Supplementary data

Supplementary data are available at www.dnaresearch.oxfordjournals.org.

## Funding

This work was supported by grants from the National Research Foundation of Korea (2015M3A9B4051044, 2010-0020259 and 2015048159, and 2014M3C9A3064548 to TYR), the POSTECH BSRI research fund (to TYR), and the BK21 PLUS fellowship program funded by the National Research Foundation of Korea (G16CN40T1201 to SIS, JP, SH, SHS, CHL and HJ). Zaa-Fok plasmid was kindly provided by Dr. Keji Zhao.

## Supplementary Material

Supplementary Data
