# Supplementary material for: Z-DNA-forming sites identified by ChIP-Seq are associated with actively transcribed regions in the human genome
Source: DNA Res. 2016 Jul 3;23(5):477–86. doi: 10.1093/dnares/dsw031 (PMC5066173; doi:10.1093/dnares/dsw031)
Supplement: Supplementary Data [file supp_23_5_477__index.html]

Z-DNA-forming sites identified by ChIP-Seq are associated with actively transcribed regions in the human genome — Supplementary Data 

# Z-DNA-forming sites identified by ChIP-Seq are associated with actively transcribed regions in the human genome

## Supplementary Data

files

- Supplementary Data - zip file
