# Supplementary material for: Z-DNA-forming sites identified by ChIP-Seq are associated with actively transcribed regions in the human genome
Source: DNA Res. 2016 Jul 3;23(5):477–86. doi: 10.1093/dnares/dsw031 (PMC5066173; doi:10.1093/dnares/dsw031)

Supplementary Fig. S1

High-throughput sequencing
Zaa rep1, rep2, rep3, IgG, input

↓

SPP peak detection using IgG as a control

↓

original replicates	rep1 vs rep2	rep1 vs rep3	rep2 vs rep3
**IDR ≤ 0.05**	197	339	427
pseudoreplicates	pr1 vs pr2 (rep1)	pr1 vs pr2 (rep2)	pr1 vs pr2 (rep3)
**IDR ≤ 0.01**	1,024	520	814
pooled-pseudoreplicates	pr1 vs pr2		
**IDR ≤ 0.001**	649		

Maximum peak number between original replicates (IDR ≤ 0.05) : 427

↓

Peaks consistent between replicates :
Top 427 peaks in the pooled replicates

↓

Removal of peaks overlapped with blacklisted regions

↓

Total 391 ZFSs

↓

Peak validation and
computational analysis

Supplementary Fig. S2

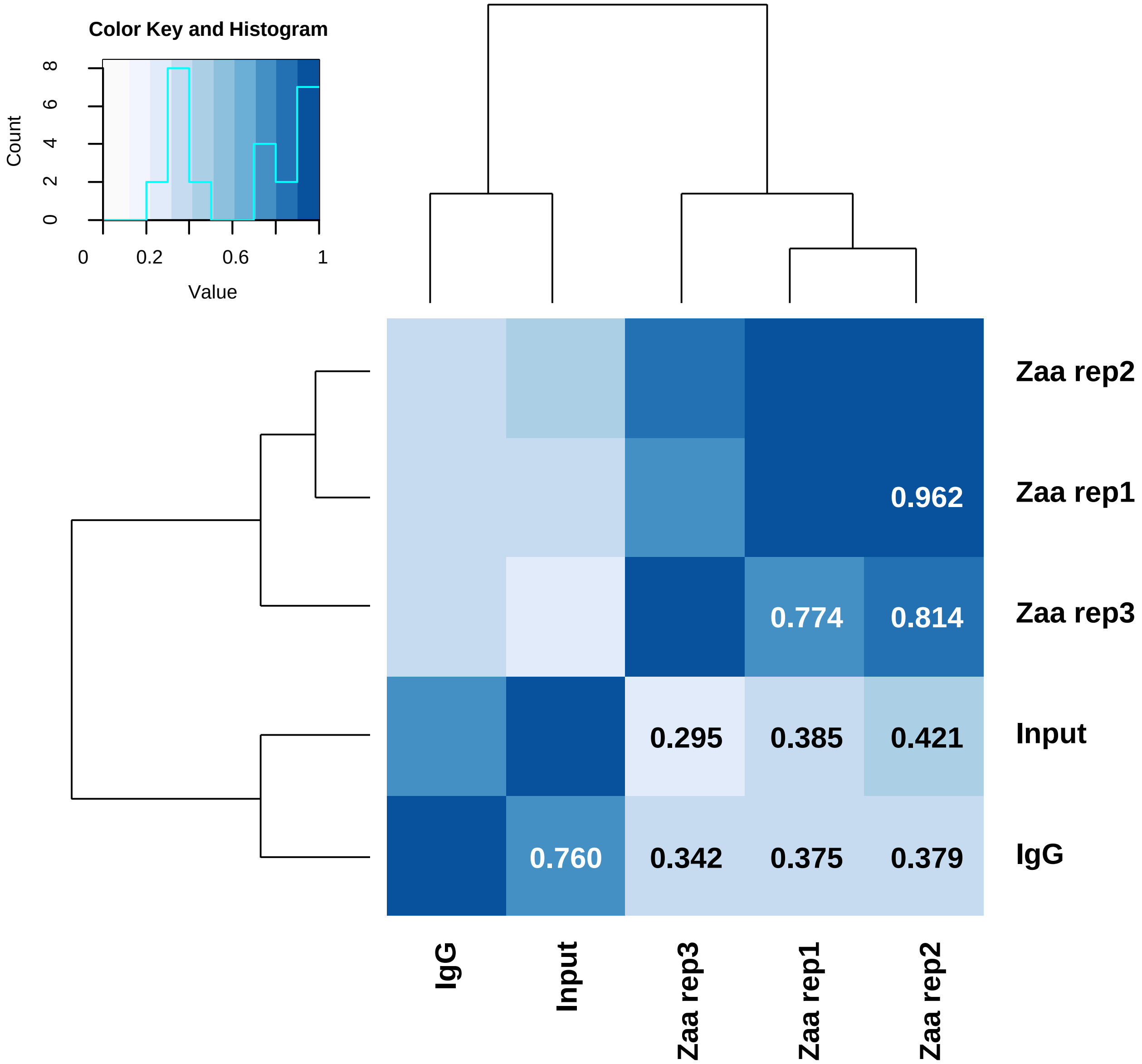

Supplementary Fig. S3

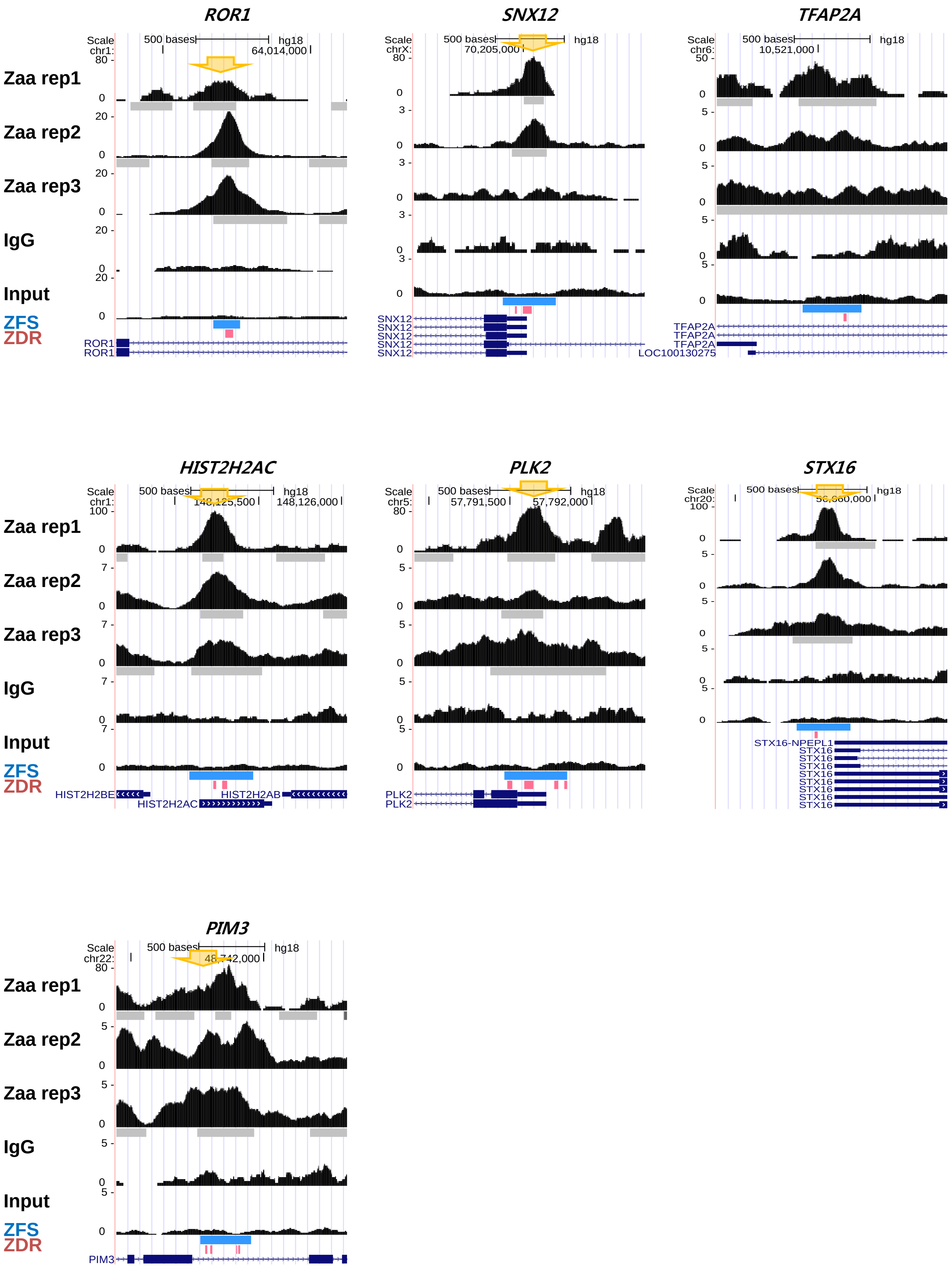

Supplementary Fig. S4

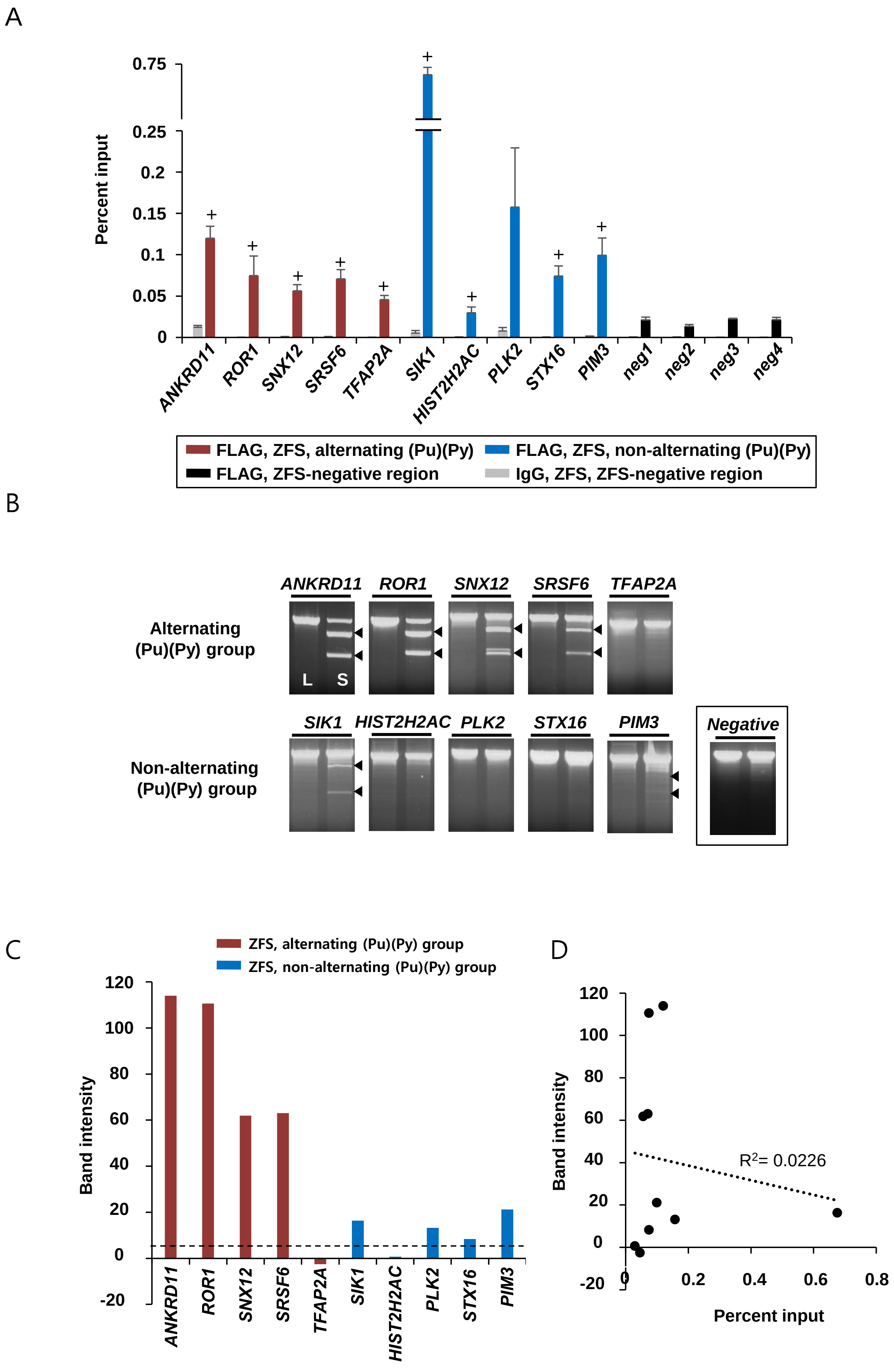

Supplementary Fig. S5

Motif logos	-log10 (p-value)	% of targets
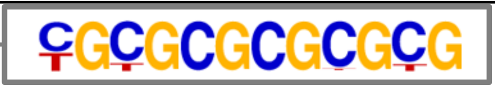	174	40.15
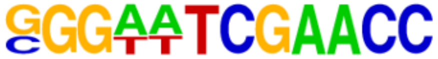	26	5.37
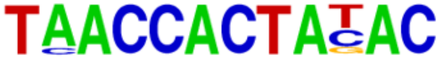	20	2.81
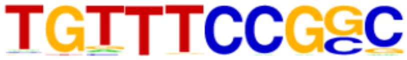	18	9.97
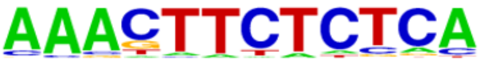	16	6.91

Similar *de novo* motifs

Motif logos	-log10 (p-value)	% of targets
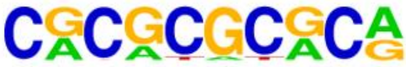	139	39.13
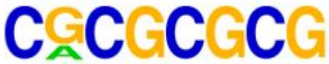	92	37.08
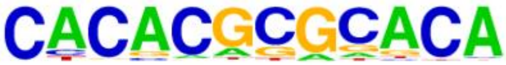	70	37.44
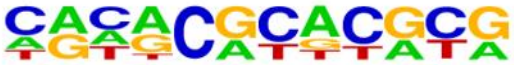	66	43.22
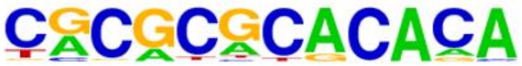	65	41.43

Supplementary Fig. S6

A

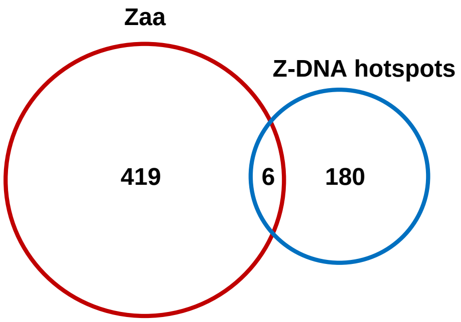

B.

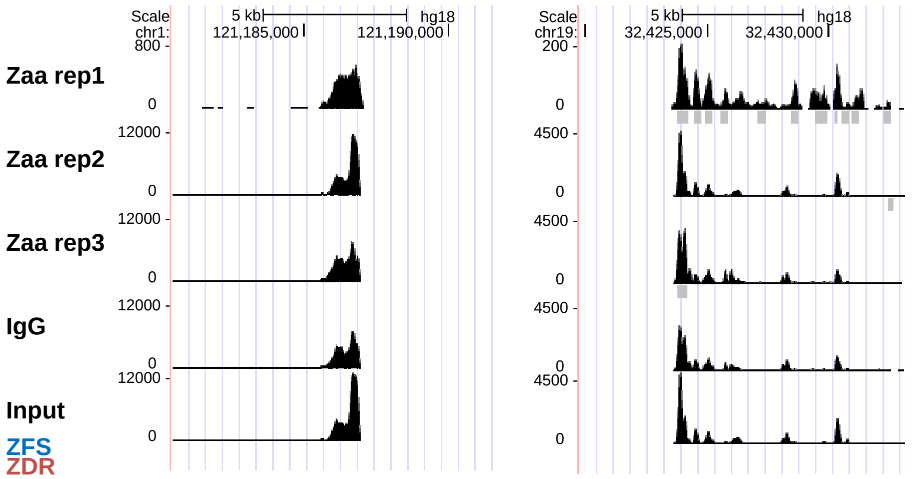

Supplementary Fig. S7

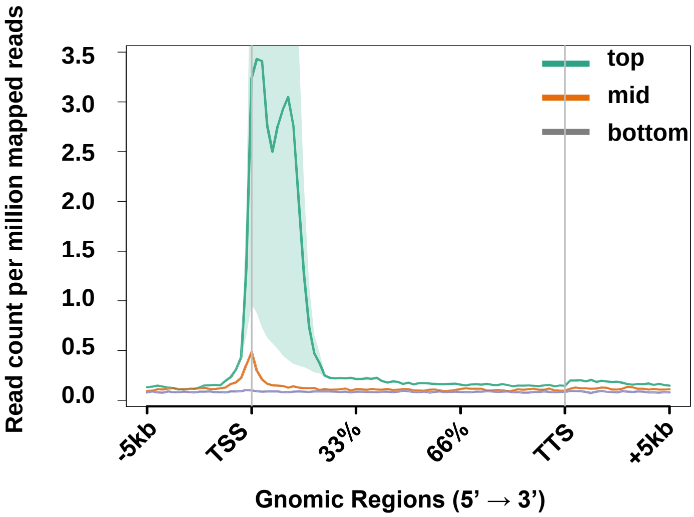

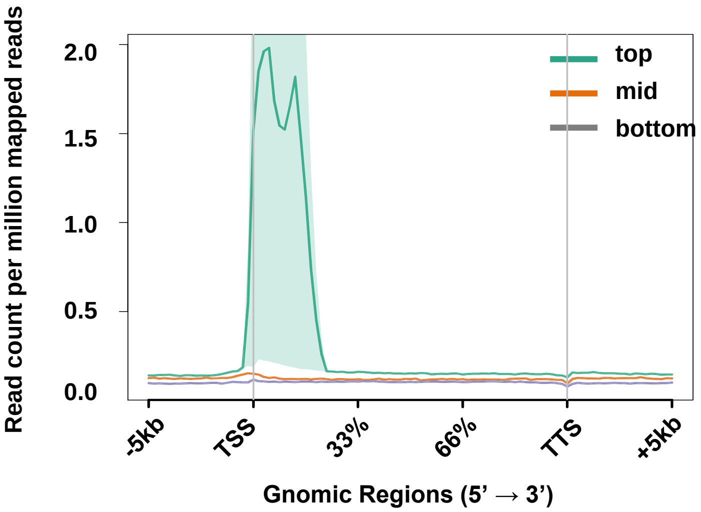

Supplementary Fig. S8

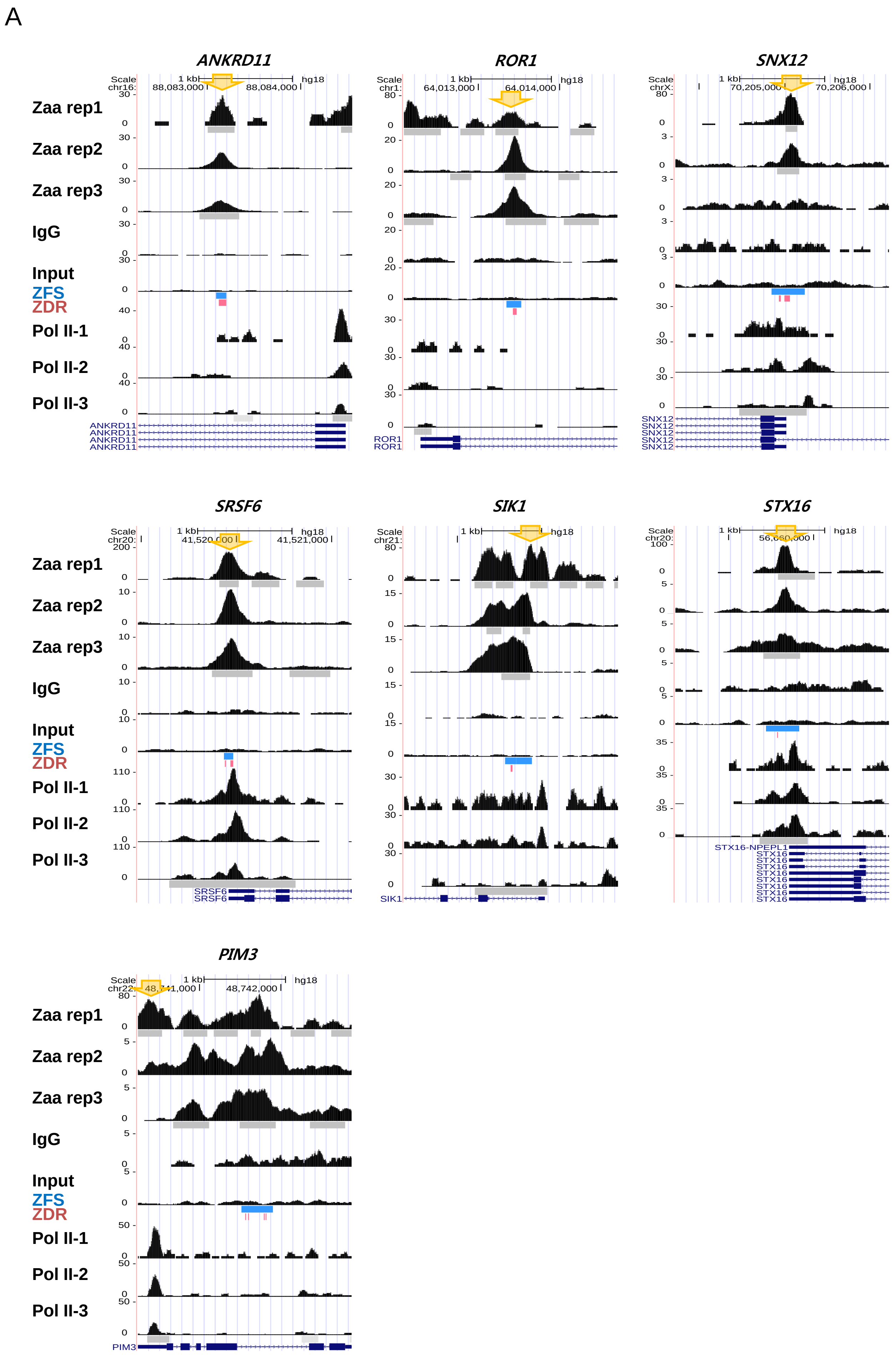

B

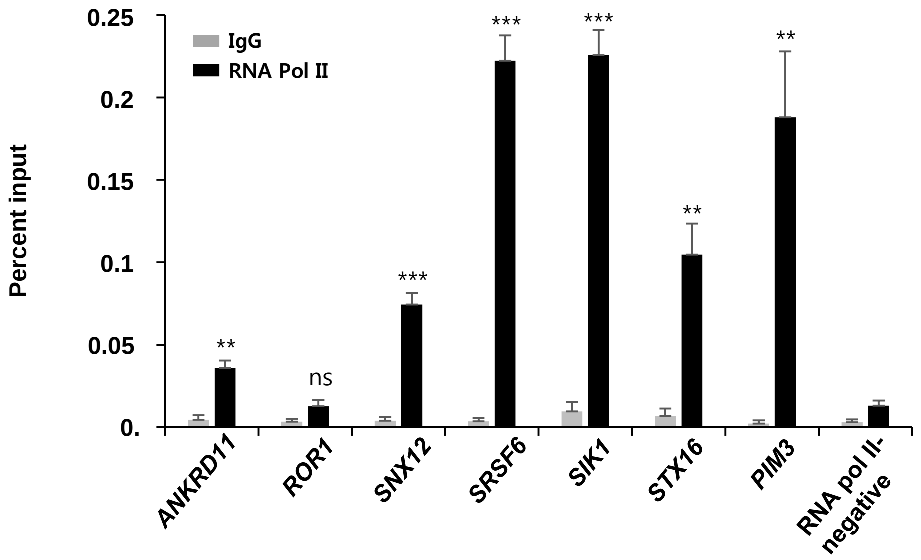

IgG
RNA Pol II
Percent input
0.25
0.2
0.15
0.1
0.05
0.
***
***
**
**
***
**
ns
ANKRD11
ROR1
SNX12
SRSF6
SIK1
STX16
PIM3
RNA pol II-negative

Supplementary Fig. S9

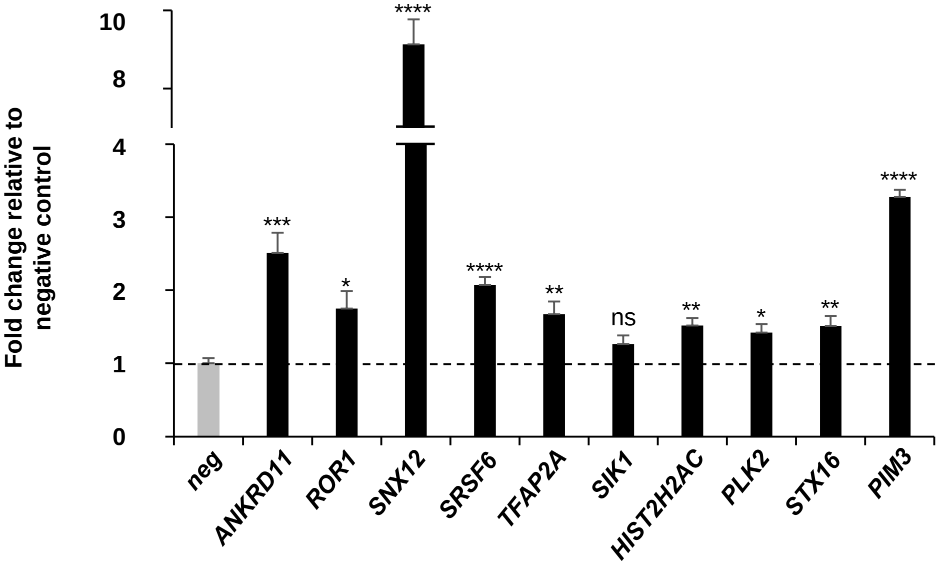

Supplement: Supplementary Data [file supp_dsw031_suppl_data.zip › SupplementaryFigures.pdf]
