# Supplementary material for: Z-DNA-forming sites identified by ChIP-Seq are associated with actively transcribed regions in the human genome
Source: DNA Res. 2016 Jul 3;23(5):477–86. doi: 10.1093/dnares/dsw031 (PMC5066173; doi:10.1093/dnares/dsw031)
Supplement: Supplementary Data [file supp_dsw031_suppl_data.zip › Supplementary_Tables.pdf]

Supplementary Table S1. Primer sequences used for Zaa or RNA polymerase II ChIP-qPCR analysis.

**Zaa ChIP-qPCR**					
	**Gene name**	**Primer sequence**			
1	*ANKRD11*	F	5'-CGACCAACCTTCCCAAATAA-3'	R	5'-CTTGGCCTCAAGGAACAATG-3'
2	*ROR1*	F	5'-CCCTCCCGAGAGTCATCATA-3'	R	5'-TAGATGGCTTTTCCCCCTCT-3'
3	*SNX12*	F	5'-CCCTCCCGCTTGCAAAATAA-3'	R	5'-CCACAAACATCACCCGGAAG-3'
4	*SRSF6*	F	5'-ACTCGCCAGTCACCATCTAG-3'	R	5'-CAAGGGCTGGTTGTCGAAC-3'
5	*TFAP2A*	F	5'-TCGAACCCACGGTCTCTATC-3'	R	5'-GCGCGGTTATTAGAAGCTCA-3'
6	*SIK1*	F	5'-CGCTGAACTCCGACATGATA-3'	R	5'-GATAGAGTGGGGGCGACAG-3'
7	*HIST2HSAC*	F	5'-GGTTCTGAGCGTTGTCTGTG-3'	R	5'-TGTTCAGTTCCTCGTCGTTG-3'
8	*PLK2*	F	5'-GGCTGGCTGGTAGGTGATAG-3'	R	5'-ATGTCGCGTATCGAGTCTCC-3'
9	*STX16*	F	5'-CTAGTGCCTGGACCCAGTTG-3'	R	5'-GCCTTCTCACTCCTCTTCTCC-3'
10	*PIM3*	F	5'-GGAGGGGCTGATGACTGTT-3'	R	5'-ACGCGATGGAGGAAGCAATA-3'
11	*FUS* 5kb up	F	5'-GTAACTTCCTGGCACGATGG-3'	R	5'-TCCGGAGTTTGCAGTTCTCT-3'
12	*SIK* 5kb up	F	5'-TCGTACCACTGCACTCCAAC-3'	R	5'-TCCTGTCAATCAAGCGAGTG-3'
13	*SRSF6* 5kb up	F	5'-TCAAGTGATCCTCCCACCTC-3'	R	5'-CAAGACCAGCCTGAACAACA-3'
14	*JUN* 10kb down	F	5'-ATGGAAACCCAGGTGTCTTG-3'	R	5'-TGTCATTTGGTGTTTCTGTGG-3'
**RNA polymerase II ChIP-qPCR**					
	**Gene name**	**Primer sequence**			
1	*SIK1*	F	5'-CGCGGACTCACTCCTACC-3'	R	5'-CAGTAGGCACCCGAGCAG-3'
2	*PIM3*	F	5'-AAGACAGGCGCCAAGCTG-3'	R	5'-CGAACTTGGAGAGCAGCATC-3'
3	RNA polII-negative	F	5'-GAACCTGGGGTGGATCATTC-3'	R	5'-TCGTAACGGGCACACATTAG-3'

Each gene labeled each ZFS. F and R indicate forward and reverse primers, respectively. The same primer pairs which were used in Zaa ChIP-qPCR were used in RNA polymerase II ChIP-qPCR except SIK1 and PIM3.

Supplementary Table S2. Statistics information of seqeuence reads.

	**Read length(bp)**	**Number of sequenced reads**	**Number of aligned reads**	**Unique positions**	**Duplication rate**	**Sequencing depth**	**NSC**	**RSC**
Zaa rep1	42	19,864,722	11,209,531	3,191,684	71.50%	0.18±3.76	1.310	1.729
Zaa rep2	2X100	189,034,392	178,755,114	168,529,761	8.16%	5.81±78.35	1.030	3.059
Zaa rep3	2X100	131,782,000	124,166,157	114,679,862	7.64%	4.03±60.59	1.047	2.992
IgG	2X100	47,211,804	44,732,898	42,996,580	4.17%	1.45±13.84	–	–
Input	2X100	217,450,246	212,253,185	198,626,530	6.42%	6.90±96.36	–	–

NSC : normalized standard coefficient, The NSC value recommended by the ECODE consortia is >1.05.

RSC : relative strand correlation. The RSC value recommended by the ENCODE consortia is >0.8.

Supplementary Table S3. List of 391 ZFSs.

Chr	Start	End	Peak Size(bp)	Distance to TSS (bp)	Gene name	Nearest promoter ID	FPKM	ZDR(-0.08)	ZDR(-0.07)	Gene description
chr17	19351954	19352338	384	-25613	SLC47A1	NM_018242	10.6223	no	no	solute carrier family 47 (multidrug and toxin extrusion), member 1
chr1	58520414	58520798	384	-31807	DAB1	NM_021080	11.1994	no	no	Dab, reelin signal transducer, homolog 1 (Drosophila)
chr17	77648469	77648853	384	734	FASN	NM_004104	251.515	yes	no	fatty acid synthase
chr8	124477661	124478045	384	33	ATAD2	NM_014109	22.9152	yes	yes	ATPase family, AAA domain containing 2
chr5	57791466	57791850	384	65	PLK2	NM_006622	26.082	yes	no	polo-like kinase 2
chr6	166342205	166342589	384	-20880	LINC00473	NR_026860	36.9754	yes	yes	long intergenic non-protein coding RNA 473
chr7	44802968	44803352	384	400	PPIA	NM_001300981	-	yes	yes	peptidylprolyl isomerase A (cyclophilin A)
chr19	63722913	63723297	384	-372	ZBTB45	NM_032792	7.45933	yes	no	zinc finger and BTB domain containing 45
chr8	71221725	71222109	384	-75801	PRDM14	NM_024504	0.0470898	yes	yes	PR domain containing 14
chr6	22396995	22397238	243	8593	PRL	NM_000948	0	yes	yes	prolactin
chr12	16937631	16938015	384	-95125	SKP1P2	NR_036619	0.040059	yes	yes	S-phase kinase-associated protein 1 pseudogene 2
chr6	26264442	26264826	384	96	HIST1H1E	NM_005321	0.232529	yes	no	histone cluster 1, H1e
chr3	150952813	150953197	384	-29	COMMD2	NM_016094	7.24253	yes	no	COMM domain containing 2
chr19	52377456	52377840	384	-44391	MIR3190	NR_036158	751.369	yes	yes	microRNA 3190
chr1	38228248	38228632	384	-92	SF3A3	NM_006802	76.8006	yes	no	splicing factor 3a, subunit 3, 60kDa
chrX	81099404	81099788	384	-755499	HMGN5	NM_030763	16.5545	yes	yes	high mobility group nucleosome binding domain 5
chr1	6721571	6721955	384	11589	LOC100505887	NR_104621	-	yes	yes	uncharacterized LOC100505887
chr5	88015363	88015747	384	821	LINC00461	NR_015436	0	yes	yes	long intergenic non-protein coding RNA 461
chr15	60424400	60424677	277	2018	MIR6085	NR_106733	-	yes	yes	microRNA 6085
chr19	46507979	46508363	384	237	CCDC97	NM_052848	14.7068	yes	yes	coiled-coil domain containing 97
chr1	213157017	213157401	384	-88299	KCNK2	NM_001017424	0.0875986	yes	yes	potassium channel, subfamily K, member 2
chr1	107919007	107919312	305	113490	VAV3	NM_001079874	1.63135	yes	yes	vav 3 guanine nucleotide exchange factor
chr11	28088198	28088582	384	2016	METTL15	NM_001297775	-	yes	no	methyltransferase like 15
chr2	200528405	200528789	384	107	TYW5	NR_109905	-	yes	no	tRNA-yW synthesizing protein 5
chr3	116884492	116884876	384	59843	GAP43	NM_001130064	2.37E-07	yes	yes	growth associated protein 43
chr17	38793886	38794270	384	27714	LINC00910	NR_027412	0.216184	yes	no	long intergenic non-protein coding RNA 910
chr7	28566779	28567329	550	-125192	CREB5	NM_001011666	9.32E-25	yes	yes	cAMP responsive element binding protein 5
chr11	117812062	117812446	384	-161	KMT2A	NM_001197104	1.41413	yes	no	lysine (K)-specific methyltransferase 2A
chr3	129024275	129024659	384	-52	MGLL	NM_001256585	4.30351	yes	yes	monoglyceride lipase
chr10	122600539	122600923	384	-50	WDR11-AS1	NR_033850	0.0877685	yes	no	WDR11 antisense RNA 1
chrX	31269735	31270119	384	-74982	DMD	NM_004016	4.82253	yes	yes	dystrophin
chr1	243093254	243093451	197	1098	HNRNPU	NM_004501	93.0109	yes	yes	heterogeneous nuclear ribonucleoprotein U (scaffold attachment factor A)
chr6	27373600	27373984	384	14198	POM121L2	NM_033482	0.0679131	yes	yes	POM121 transmembrane nucleoporin-like 2
chr2	58508192	58508576	384	-93008	LINC01122	NR_033873	0.00820092	yes	yes	long intergenic non-protein coding RNA 1122
chr11	64619994	64620378	384	23	VPS51	NM_013265	51.4715	yes	no	vacuolar protein sorting 51 homolog (S. cerevisiae)
chr6	167288706	167289090	384	1169	RNASET2	NM_003730	24.3295	yes	yes	ribonuclease T2
chr3	185986330	185986714	384	-12885	LOC101928992	NR_110043	-	yes	yes	uncharacterized LOC101928992
chr1	93317132	93317516	384	-56	MTF2	NM_001164391	5.57E-05	yes	no	metal response element binding transcription factor 2
chr5	36550367	36550751	384	-91655	SLC1A3	NM_001166696	0.231772	no	no	solute carrier family 1 (glial high affinity glutamate transporter), member 3
chr6	26379225	26379609	384	174	HIST1H3G	NM_003534	1.0368	yes	no	histone cluster 1, H3g
chr6	3963623	3964007	384	-2753	PRPF4B	NM_003913	6.8855	yes	yes	pre-mRNA processing factor 4B
chr21	17748808	17749192	384	15921	C21orf37	NR_037585	0	no	no	chromosome 21 open reading frame 37
chr11	7965084	7965468	384	-167	EIF3F	NM_003754	198.353	no	no	eukaryotic translation initiation factor 3, subunit F
chr18	66023825	66024209	384	-75	RTTN	NM_173630	3.57293	yes	no	rotatin
chr8	121526402	121526786	384	-225	MTBP	NM_022045	3.42369	no	no	MDM2 binding protein
chr11	67528068	67528452	384	-89	UNC93B1	NM_030930	24.3623	yes	yes	unc-93 homolog B1 (C. elegans)
chr1	145479251	145479635	384	-452	BCL9	NM_004326	4.87287	yes	yes	B-cell CLL/lymphoma 9
chrX	128805679	128806082	403	-289	ZDHHC9	NM_016032	12.6573	yes	yes	zinc finger, DHHC-type containing 9
chr14	34901716	34902100	384	41803	NFKBIA	NM_020529	23.1435	yes	yes	nuclear factor of kappa light polypeptide gene enhancer in B-cells inhibitor, alpha
chr10	23785769	23786153	384	17757	OTUD1	NM_001145373	3.52	yes	yes	OTU deubiquitinase 1
chrX	73080616	73081000	384	-76	JPX	NR_024582	2.76564	no	no	JPX transcript, XIST activator (non-protein coding)
chr1	143733529	143733913	384	17631	PDE4DIP	NM_001198832	0.535115	no	no	phosphodiesterase 4D interacting protein
chr12	131915397	131915781	384	-90	GOLGA3	NM_001172557	4.22285	yes	yes	golgin A3
chr1	229828872	229829256	384	-120	DISC1	NM_001164544	1.82E-49	yes	yes	disrupted in schizophrenia 1
chr12	54510683	54511067	384	-1188	DNAJC14	NM_032364	27.6844	yes	yes	DnaJ (Hsp40) homolog, subfamily C, member 14
chr2	8378637	8379021	384	7171	LINC00299	NR_034135	0	yes	yes	long intergenic non-protein coding RNA 299
chr6	12395799	12396183	384	-2524	EDN1	NM_001168319	6.32E-05	yes	yes	endothelin 1
chr7	73226330	73226714	384	-120	EIF4H	NM_031992	232.9	yes	yes	eukaryotic translation initiation factor 4H
chr5	58203782	58204166	384	127542	PDE4D	NM_001197223	0.0587752	no	no	phosphodiesterase 4D, cAMP-specific
chr16	86451896	86452280	384	8513	SLC7A5	NM_003486	549.537	yes	yes	solute carrier family 7 (amino acid transporter light chain, L system), member 5
chr1	118429479	118429863	384	99700	SPAG17	NM_206996	0.0145399	yes	yes	sperm associated antigen 17
chr1	143807572	143807956	384	0	SEC22B	NM_004892	65.7576	no	no	SEC22 vesicle trafficking protein homolog B (S. cerevisiae) (gene/pseudogene)
chr17	27693141	27693525	384	8	C17orf75	NM_022344	3.09077	yes	no	chromosome 17 open reading frame 75
chr1	144108051	144108435	384	-16305	HFE2	NM_213653	1.69E-05	no	no	hemochromatosis type 2 (juvenile)
chr5	4874044	4874428	384	47642	LOC101929153	NR_104619	-	yes	no	uncharacterized LOC101929153
chr10	42597976	42598360	384	208	BMS1	NM_014753	12.151	yes	no	BMS1 ribosome biogenesis factor
chrX	73564139	73564523	384	6278	SLC16A2	NM_006517	0.0511449	yes	yes	solute carrier family 16, member 2 (thyroid hormone transporter)
chr9	97311539	97311923	384	-1079	PTCH1	NM_000264	0.690446	yes	yes	patched 1
chr20	26137404	26137788	384	273	MIR663AHG	NR_040095	0.0333671	yes	no	MIR663A host gene (non-protein coding)
chr4	106849111	106849495	384	27	INTS12	NM_001142471	8.4077	no	no	integrator complex subunit 12
chr9	101623601	101623985	384	-165	NR4A3	NM_173199	2.38E-07	yes	yes	nuclear receptor subfamily 4, group A, member 3
chr14	99374916	99375069	153	45494	EML1	NM_001008707	0.000123239	yes	yes	echinoderm microtubule associated protein like 1
chr12	12769392	12769776	384	-534	APOLD1	NM_001130415	0.940069	yes	yes	apolipoprotein L domain containing 1
chr6	21701493	21701877	384	-266	SOX4	NM_003107	15.7575	yes	yes	SRY (sex determining region Y)-box 4
chr2	233095558	233095942	384	2333	PRSS56	NM_001195129	0.464478	yes	no	protease, serine, 56
chr21	43670955	43671215	260	345	SIK1	NM_173354	105.685	no	no	salt-inducible kinase 1
chr21	25901510	25901894	384	-30	MRPL39	NM_080794	1.24E-05	yes	no	mitochondrial ribosomal protein L39
chr2	112749286	112749670	384	-171	ZC3H6	NM_198581	0.215976	no	no	zinc finger CCCH-type containing 6
chr1	71460923	71461307	384	141520	ZRANB2-AS2	NR_046217	0	no	no	ZRANB2 antisense RNA 2 (head to head)
chr6	26646098	26646482	384	-261	HMGN4	NM_006353	25.5278	yes	yes	high mobility group nucleosomal binding domain 4
chr19	16867576	16867866	290	6895	F2RL3	NM_003950	0.0132886	yes	yes	coagulation factor II (thrombin) receptor-like 3
chr12	123965117	123965501	384	231	UBC	NM_021009	476.306	no	no	ubiquitin C
chr8	24870493	24870877	384	-385	NEFL	NM_006158	0.00922811	yes	yes	neurofilament, light polypeptide
chr18	143224	143608	384	-5067	USP14	NM_001037334	0.301415	yes	yes	ubiquitin specific peptidase 14 (tRNA-guanine transglycosylase)
chr7	36684564	36684948	384	45923	AOAH	NM_001177506	1.77E-08	yes	yes	acyloxyacyl hydrolase (neutrophil)
chr20	41519866	41519966	100	-2	SRSF6	NM_006275	33.9554	yes	yes	serine/arginine-rich splicing factor 6
chr21	43664594	43664978	384	6644	SIK1	NM_173354	105.685	yes	no	salt-inducible kinase 1
chr1	144110428	144110812	384	-13928	HFE2	NM_213653	1.69E-05	no	no	hemochromatosis type 2 (juvenile)
chr2	40202660	40203044	384	204574	SLC8A1-AS1	NR_038441	0	yes	yes	SLC8A1 antisense RNA 1
chr15	64781145	64781529	384	-391	SMAD6	NR_027654	0.656805	yes	yes	SMAD family member 6
chr6	50902019	50902403	384	7813	TFAP2B	NM_003221	0.0120549	yes	yes	transcription factor AP-2 beta (activating enhancer binding protein 2 beta)
chr1	23758380	23758764	384	300	ID3	NM_002167	192.94	yes	yes	inhibitor of DNA binding 3, dominant negative helix-loop-helix protein
chr1	203357869	203358253	384	-288	RBBP5	NM_001193272	1.95986	yes	no	retinoblastoma binding protein 5
chr2	226193208	226193592	384	-143005	MIR548AR	NR_049839	0	yes	yes	microRNA 548ar
chr20	25571059	25571443	384	-18603	NANP	NM_152667	2.59542	yes	yes	N-acetylneuraminic acid phosphatase
chrX	50231186	50231570	384	-901	DGKK	NM_001013742	0.00903413	yes	yes	diacylglycerol kinase, kappa
chr1	86943102	86943486	384	453	SH3GLB1	NM_001206653	2.05E-09	yes	no	SH3-domain GRB2-like endophilin B1
chr6	35335203	35335587	384	-74	ZNF76	NM_003427	12.5196	yes	yes	zinc finger protein 76
chr1	178118303	178118687	384	695	TOR1AIP1	NM_001267578	4.22925	yes	no	torsin A interacting protein 1
chr12	123529844	123530228	384	87927	NCOR2	NM_006312	33.861	yes	yes	nuclear receptor corepressor 2
chr5	40870983	40871367	384	-31	RPL37	NM_000997	825.865	no	no	ribosomal protein L37
chr12	123990003	123990627	624	24269	MIR5188	NR_049820	0	no	no	microRNA 5188
chr6	26139984	26140368	384	91	HIST1H3B	NM_003537	0.127142	yes	no	histone cluster 1, H3b
chr2	9523794	9524178	384	-8135	IAH1	NM_001039613	20.2771	yes	yes	isoamyl acetate-hydrolyzing esterase 1 homolog (S. cerevisiae)

Chr	Start	End	Peak Size(bp)	Distance to TSS (bp)	Gene name	Nearest promoter ID	FPKM	ZDR(-0.08)	ZDR(-0.07)	Gene description
chr7	70828169	70828553	384	-417669	MIR3914-1	NR_037477	0	yes	yes	microRNA 3914-1
chr17	46585707	46586091	384	3	NME1-NME2	NR_037149	0.071663	yes	no	NME1-NME2 readthrough
chr6	20970712	20971096	384	328237	CDKAL1	NM_017774	4.42019	yes	yes	CDK5 regulatory subunit associated protein 1-like 1
chr6_random	162201	162585	384	504093	LOC401286	NR_117091	-	yes	no	uncharacterized LOC401286
chr11	77468422	77468806	384	299	NDUFC2-KCTD14	NM_001203260	4.20E-07	yes	no	NDUFC2-KCTD14 readthrough
chr16	88083094	88083199	105	1324	ANKRD11	NR_045839	5.11787	yes	yes	ankyrin repeat domain 11
chr1	85563434	85563818	384	-48451	BCL10	NM_003921	2.77844	yes	yes	B-cell CLL/lymphoma 10
chr2	232283058	232283442	384	1771	PTMA	NM_001099285	125.131	yes	yes	prothymosin, alpha
chr7	130802052	130802436	384	89672	PODXL	NM_005397	94.6994	yes	yes	podocalyxin-like
chr1	165950417	165950801	384	-7202	MPZL1	NM_024569	4.76805	no	no	myelin protein zero-like 1
chr7	101562134	101562518	384	-152747	SH2B2	NM_020979	1.98869	yes	yes	SH2B adaptor protein 2
chr1	233900104	233900488	384	-19619	GNG4	NM_001098721	4.76E-05	no	no	guanine nucleotide binding protein (G protein), gamma 4
chr5	36987750	36988134	384	75324	NIPBL	NM_133433	13.9417	yes	yes	Nipped-B homolog (Drosophila)
chr19	1206122	1206506	384	6762	MIDN	NM_177401	16.6974	yes	no	midnolin
chr4	72240959	72241343	384	-30716	SLC4A4	NM_001134742	1.16E-05	yes	yes	solute carrier family 4 (sodium bicarbonate cotransporter), member 4
chr4	160436181	160436565	384	27925	RAPGEF2	NM_014247	5.63809	yes	yes	Rap guanine nucleotide exchange factor (GEF) 2
chr1	179258575	179258959	384	113	STX6	NM_005819	11.2112	yes	no	syntaxin 6
chr5	26070249	26070633	384	1004005	CDH9	NM_016279	0.011694	yes	yes	cadherin 9, type 2 (T1-cadherin)
chr6	111242938	111243322	384	82	CDK19	NM_001300960	-	yes	yes	cyclin-dependent kinase 19
chr3	171238939	171239323	384	702	GPR160	NM_014373	0.427238	yes	yes	G protein-coupled receptor 160
chr5	140085800	140086184	384	64	VTRNA1-3	NR_026705	0	yes	no	vault RNA 1-3
chr15	62466879	62467263	384	15	TRIP4	NM_016213	8.76092	yes	no	thyroid hormone receptor interactor 4
chr9	111918289	111918673	384	-9121	AKAP2	NM_001136562	5.24836	yes	yes	A kinase (PRKA) anchor protein 2
chr17	54111741	54112125	384	12482	TEX14	NM_001201457	2.83E-05	no	no	testis expressed 14
chr12	94953789	94954173	384	-411	LTA4H	NM_000895	40.5048	no	no	leukotriene A4 hydrolase
chr1	143699435	143699819	384	6763	PDE4DIP	NM_001002810	8.14E-257	yes	no	phosphodiesterase 4D interacting protein
chr11	57265223	57265607	384	117	C11orf31	NM_170746	34.058	no	no	chromosome 11 open reading frame 31
chr12	14815011	14815395	384	129	HIST4H4	NM_175054	1.31659	yes	no	histone cluster 4, H4
chr1	144094028	144094412	384	-30328	HFE2	NM_213653	1.69E-05	yes	no	hemochromatosis type 2 (juvenile)
chr3	129688167	129688551	384	1095	GATA2	NM_001145662	6.66081	yes	yes	GATA binding protein 2
chr20	34144836	34145220	384	982	EPB41L1	NM_177996	22.4756	yes	yes	erythrocyte membrane protein band 4.1-like 1
chr15	47501480	47501864	384	-995	FGF7	NM_002009	0.0134894	yes	yes	fibroblast growth factor 7
chr17	12440988	12441372	384	47170	LINC00670	NR_034144	0.0279426	yes	yes	long intergenic non-protein coding RNA 670
chr1	100207928	100208312	384	187	SLC35A3	NM_001271684	1.15E-05	yes	no	solute carrier family 35 (UDP-N-acetylglucosamine (UDP-GlcNAc) transporter), member A3
chr2	232282254	232282638	384	967	PTMA	NM_001099285	125.131	yes	no	prothymosin, alpha
chr19	60383275	60383659	384	65	SYT5	NM_003180	0.0414714	yes	yes	synaptotagmin V
chr6	26164381	26164765	384	105	HIST1H1C	NM_005319	20.9332	yes	yes	histone cluster 1, H1c
chr6	27215111	27215495	384	236	HIST1H4I	NM_003495	2.48273	yes	no	histone cluster 1, H4i
chr2	235791491	235791767	276	266262	SH3BP4	NM_014521	19.7982	yes	yes	SH3-domain binding protein 4
chr3	11389125	11389509	384	100307	ATG7	NM_001144912	5.02E-05	yes	yes	autophagy related 7
chr5	129926680	129926812	132	602194	HINT1	NR_073488	2.79476	yes	yes	histidine triad nucleotide binding protein 1
chr9	112250061	112250445	384	-110268	TXNDC8	NM_001286947	-	yes	yes	thioredoxin domain containing 8 (spermatozoa)
chr17	18149353	18149737	384	9501	TOP3A	NM_004618	13.8374	yes	yes	topoisomerase (DNA) III alpha
chr1	90243158	90243542	384	10084	ZNF326	NM_182976	13.8166	no	no	zinc finger protein 326
chr12	43628693	43629077	384	-34907	NELL2	NM_001145110	5.52234	yes	no	NEL-like 2 (chicken)
chr10	57800510	57800894	384	-9662	ZWINT	NM_032997	6.60988	no	no	ZW10 interacting kinetochore protein
chr1	71584028	71584412	384	264625	ZRANB2-AS2	NR_046217	0	yes	no	ZRANB2 antisense RNA 2 (head to head)
chr2	11523639	11524023	384	-77	E2F6	NM_001278277	-	yes	yes	E2F transcription factor 6
chr15	36533446	36533830	384	18	FAM98B	NM_173611	11.8417	no	no	family with sequence similarity 98, member B
chr6	8380235	8380619	384	372	SLC35B3	NM_001142541	1.04691	yes	yes	solute carrier family 35 (adenosine 3'-phospho 5'-phosphosulfate transporter), member B3
chr16	15929960	15930344	384	-20783	ABCC1	NM_004996	49.8092	yes	yes	ATP-binding cassette, sub-family C (CFTR/MRP), member 1
chr9	37790579	37790963	384	-12	DCAF10	NM_024345	5.87752	no	no	DDB1 and CUL4 associated factor 10
chr6	10520900	10521284	384	-499	TFAP2A	NM_001032280	4.21434	yes	no	transcription factor AP-2 alpha (activating enhancer binding protein 2 alpha)
chr5	167845103	167845487	384	-746	RARS	NM_002887	52.8986	yes	yes	arginyl-tRNA synthetase
chr2	241690277	241690661	384	-49	MTERF4	NM_182501	11.5223	yes	yes	mitochondrial transcription termination factor 4
chr12	63321284	63321668	384	30916	RASSF3	NM_178169	11.5199	yes	yes	Ras association (RalGDS/AF-6) domain family member 3
chr17	53439533	53439917	384	-19	SRSF1	NM_006924	62.6089	yes	yes	serine/arginine-rich splicing factor 1
chr1	146273121	146273505	384	-11	MIR5087	NR_049810	0	no	no	microRNA 5087
chr16	71646913	71647297	384	2930	ZFHX3	NM_001164766	1.9769	yes	yes	zinc finger homeobox 3
chr3	197098927	197099311	384	-5456	MIR6829	NR_106887	-	yes	yes	microRNA 6829
chr1	211031646	211032030	384	45	TATDN3	NM_001146170	0.170272	yes	no	TatD DNase domain containing 3
chr1	148474935	148475052	117	135	ANP32E	NM_030920	42.4604	yes	yes	acidic (leucine-rich) nuclear phosphoprotein 32 family, member E
chr5	17271479	17271863	384	1001	BASP1	NM_006317	275.651	no	no	brain abundant, membrane attached signal protein 1
chr6	27208667	27209051	384	63	HIST1H2AG	NM_021064	4.16629	yes	no	histone cluster 1, H2ag
chr8	95801033	95801417	384	-54	DPY19L4	NM_181787	3.84944	yes	no	dpy-19-like 4 (C. elegans)
chr2	122004810	122005194	384	76	RNU4ATAC	NR_023343	0	yes	no	RNA, U4atac small nuclear (U12-dependent splicing)
chr7	137862889	137863273	384	67462	TRIM24	NM_015905	7.59527	yes	no	tripartite motif containing 24
chr22	17799809	17800193	384	-35	MRPL40	NM_003776	42.6098	yes	no	mitochondrial ribosomal protein L40
chr1	202932361	202932745	384	-11333	LRRN2	NM_201630	0.594868	yes	yes	leucine rich repeat neuronal 2
chr1	143811422	143811806	384	3850	SEC22B	NM_004892	65.7576	no	no	SEC22 vesicle trafficking protein homolog B (S. cerevisiae) (gene/pseudogene)
chr3	71715315	71715699	384	87	FOXP1	NM_001244808	4.00365	yes	yes	forkhead box P1
chr8	100094283	100094667	384	-195	VPS13B	NM_181661	2.23E-12	no	no	vacuolar protein sorting 13 homolog B (yeast)
chr7	134997709	134998093	384	140	C7orf73	NM_001130929	12.6643	no	no	chromosome 7 open reading frame 73
chr3	51547522	51547906	384	-2922	RAD54L2	NM_015106	5.92672	yes	yes	RAD54-like 2 (S. cerevisiae)
chr16	1954465	1954849	384	171	RPS2	NM_002952	2954.62	yes	no	ribosomal protein S2
chr15	73074765	73075149	384	28	SCAMP5	NM_001178112	0.000188903	yes	yes	secretory carrier membrane protein 5
chr5	10643482	10643866	384	26239	ANKRD33B	NM_001164440	1.93244	yes	yes	ankyrin repeat domain 33B
chr7	138675823	138676207	384	279	C7orf55	NR_073059	1.74409	yes	yes	chromosome 7 open reading frame 55
chr1	77921499	77921883	384	-760	ZZZ3	NM_015534	11.3449	yes	no	zinc finger, ZZ-type containing 3
chr1	143245325	143245709	384	-59435	NBPF12	NM_001278141	-	yes	no	neuroblastoma breakpoint family, member 12
chr1	143739564	143739948	384	11596	PDE4DIP	NM_001198832	0.535115	no	no	phosphodiesterase 4D interacting protein
chr21	45353448	45353832	384	34719	ADARB1	NM_015834	3.97441	yes	yes	adenosine deaminase, RNA-specific, B1
chr1	44003869	44004253	384	58270	ST3GAL3	NM_174967	2.68E-37	yes	yes	ST3 beta-galactoside alpha-2,3-sialyltransferase 3
chr1	85373052	85373436	384	1179	MIR4423	NR_039619	0	no	no	microRNA 4423
chr6	26307112	26307496	384	196	HIST1H3D	NM_003530	0.931419	yes	no	histone cluster 1, H3d
chr12	30742735	30743119	384	-2731	IPO8	NM_006390	21.0371	yes	yes	importin 8
chr12	119213833	119214217	384	-10482	SIRT4	NM_012240	0.33467	yes	no	sirtuin 4
chr1	44959796	44960180	384	-18089	KIF2C	NM_006845	41.9462	no	no	kinesin family member 2C
chr1	67313970	67314354	384	-21494	SLC35D1	NM_015139	3.21731	no	no	solute carrier family 35 (UDP-GlcA/UDP-GalNAc transporter), member D1
chr5	17269989	17270373	384	249	BASP1	NM_001271606	455.997	no	no	brain abundant, membrane attached signal protein 1
chr6	40662967	40663351	384	-55	LRFN2	NM_020737	0	yes	yes	leucine rich repeat and fibronectin type III domain containing 2
chr12	46785907	46786291	384	-57	SENP1	NR_051992	0.000417778	yes	no	SUMO1/sentrin specific peptidase 1
chr2	233096693	233097077	384	-2229	CHRND	NR_046334	0.00564681	yes	yes	cholinergic receptor, nicotinic, delta (muscle)
chr1	66196847	66197231	384	-33848	PDE4B	NM_001037340	0.704632	yes	yes	phosphodiesterase 4B, cAMP-specific
chr11	43290039	43290423	384	150	API5	NM_006595	10.7437	yes	no	apoptosis inhibitor 5
chr6	27306138	27306522	384	-17151	PRSS16	NM_005865	0.239748	yes	no	protease, serine, 16 (thymus)
chr1	45985174	45985558	384	3706	IPP	NM_001145349	0.526836	yes	yes	intracisternal A particle-promoted polypeptide
chr1	203447165	203447549	384	-7	DSTYK	NM_199462	0.455899	no	no	dual serine/threonine and tyrosine protein kinase
chr5	26528893	26529277	384	545361	CDH9	NM_016279	0.011694	no	no	cadherin 9, type 2 (T1-cadherin)
chr13	40243132	40243516	384	23	MRPS31	NM_005830	7.05638	yes	no	mitochondrial ribosomal protein S31
chrX	18912748	18913132	384	-539	PHKA2	NM_000292	7.63923	no	no	phosphorylase kinase, alpha 2 (liver)
chr4	40627072	40627456	384	-73298	APBB2	NM_001166053	1.76E-32	yes	no	amyloid beta (A4) precursor protein-binding, family B, member 2
chr15	29184810	29185194	384	-3781	TRPM1	NM_002420	0.0058344	yes	yes	transient receptor potential cation channel, subfamily M, member 1
chr2	3601008	3601392	384	472	RPS7	NM_001011	1055.33	yes	no	ribosomal protein S7
chr17	7938130	7938514	384	6223	MIR4314	NR_036201	0	yes	yes	microRNA 4314

Chr	Start	End	Peak Size(bp)	Distance to TSS (bp)	Gene name	Nearest promoter ID	FPKM	ZDR(-0.08)	ZDR(-0.07)	Gene description
chr7	44496655	44497039	384	63	NUDCD3	NM_015332	25.6083	yes	no	NudC domain containing 3
chr2	47257177	47257561	384	-125	CALM2	NM_001743	203.834	no	no	calmodulin 2 (phosphorylase kinase, delta)
chr1	247134647	247135031	384	-14901	ZNF692	NM_001193328	0.645351	no	no	zinc finger protein 692
chr1	159664328	159664712	384	-60223	C1orf192	NM_001013625	6.04479	no	no	chromosome 1 open reading frame 192
chr8	96350891	96351275	384	-445	C8orf37	NM_177965	1.44062	yes	no	chromosome 8 open reading frame 37
chr6_random	1293528	1293912	384	251687	TMEM242	NM_018452	0	yes	no	transmembrane protein 242
chr7	38754169	38754553	384	-61147	FAM183B	NR_028347	0	yes	yes	acyloxyacyl hydrolase (neutrophil)
chr2	61097755	61098139	384	-369	PEX13	NM_002618	4.28515	yes	no	peroxisomal biogenesis factor 13
chr5	43519457	43519841	384	100	C5orf28	NM_022483	8.3249	yes	no	chromosome 5 open reading frame 28
chr1	18924893	18925277	384	94998	PAX7	NM_002584	0.014727	yes	yes	paired box 7
chr4	24834480	24834864	384	-10079	PI4K2B	NM_018323	5.53331	no	no	phosphatidylinositol 4-kinase type 2 beta
chr5	58370627	58371011	384	277	PDE4D	NM_001197221	0.441299	yes	no	phosphodiesterase 4D, cAMP-specific
chr22	38246364	38246748	384	41	ATF4	NM_182810	444.51	yes	yes	activating transcription factor 4
chr3	48104029	48104413	384	1552	MAP4	NM_030885	0.0247037	yes	yes	microtubule-associated protein 4
chr3	41215705	41216089	384	-49	CTNNB1	NM_001904	50.8072	yes	no	catenin (cadherin-associated protein), beta 1, 88kDa
chr9	107046308	107046692	384	-215	SLC44A1	NM_080546	9.31772	yes	no	solute carrier family 44 (choline transporter), member 1
chr16	79668383	79668767	384	-202	C16orf46	NM_152337	1.62E-06	yes	no	chromosome 16 open reading frame 46
chr21	33065963	33066347	384	-115	PAXBP1	NM_016631	2.59443	yes	yes	PAX3 and PAX7 binding protein 1
chr18	6422004	6422388	384	-17286	L3MBTL4	NM_173464	0	yes	yes	l(3)mbt-like 4 (Drosophila)
chr19	632331	632715	384	5134	FSTL3	NM_005860	90.057	yes	no	follistatin-like 3 (secreted glycoprotein)
chr1	181707627	181708011	384	-79	SMG7-AS1	NR_040063	0.193888	yes	no	SMG7 antisense RNA 1
chr1	85754616	85755000	384	-51331	DDAH1	NM_012137	7.83137	no	no	dimethylarginine dimethylaminohydrolase 1
chr6_random	1286803	1287187	384	258412	TMEM242	NM_018452	0	yes	no	transmembrane protein 242
chr1	70316869	70317253	384	126888	LRRC40	NM_017768	7.43542	yes	yes	leucine rich repeat containing 40
chr1	68071618	68072002	384	-67	GNG12	NM_018841	30.5407	yes	no	guanine nucleotide binding protein (G protein), gamma 12
chr6	27222836	27223220	384	188	HIST1H2AH	NM_080596	0.435997	yes	no	histone cluster 1, H2ah
chr1	247166994	247167378	384	121	PGBD2	NM_170725	1.83875	yes	no	piggyBac transposable element derived 2
chr20	56659442	56659826	384	-81	STX16	NR_037943	1.21935	yes	no	syntaxin 16
chr10	101978545	101978929	384	597	CHUK	NM_001278	7.54234	yes	yes	conserved helix-loop-helix ubiquitous kinase
chr11	69984163	69984547	384	62095	CTTN	NM_138565	0.000139411	yes	yes	cortactin
chr19	48772177	48772561	384	-423	PINLYP	NM_001193621	0.398506	yes	yes	phospholipase A2 inhibitor and LY6/PLAUR domain containing
chr1	85777671	85778055	384	38771	DDAH1	NM_001134445	1.45615	no	no	dimethylarginine dimethylaminohydrolase 1
chr12	97421270	97421654	384	302	LOC643770	NR_038383	0.124414	no	no	uncharacterized LOC643770
chr6	26128606	26128990	384	101	HIST1H3A	NM_003529	0.128606	yes	no	histone cluster 1, H3a
chr1	146202374	146202758	384	-16600	LOC102724558	NR_120328	-	no	no	uncharacterized LOC102724558
chr1	146607964	146608348	384	-38996	PPIAL4D	NM_001164261	0.152113	yes	no	peptidylprolyl isomerase A (cyclophilin A)-like 4D
chr6	10518124	10518508	384	-2221	TFAP2A-AS1	NR_033910	1.59098	yes	yes	TFAP2A antisense RNA 1
chr8	146248537	146248921	384	101	C8orf33	NM_023080	85.0361	yes	no	chromosome 8 open reading frame 33
chr5	138657857	138658241	384	711	MATR3	NM_001194956	1.9148	yes	yes	matrin 3
chr8	81567804	81568188	384	6993	ZBTB10	NM_001105539	1.84991	yes	yes	zinc finger and BTB domain containing 10
chr13	20989756	20990140	384	-58439	ZDHHC20	NR_104486	-	yes	yes	zinc finger, DHHC-type containing 20
chr19	13124876	13125260	384	2786	IER2	NM_004907	162.025	yes	no	immediate early response 2
chr9	69885660	69886044	384	-155821	CBWD5	NM_001286835	-	yes	yes	COBW domain containing 5
chr19	12763234	12763618	384	116	JUNB	NM_002229	150.883	yes	no	jun B proto-oncogene
chr1	64013342	64013523	181	1154	ROR1	NM_001083592	0.418379	yes	yes	receptor tyrosine kinase-like orphan receptor 1
chr11	62365477	62365861	384	-1465	WDR74	NM_018093	67.383	no	no	WD repeat domain 74
chr4	4594510	4594894	384	-26	STX18	NM_016930	17.7706	yes	no	syntaxin 18
chr7	40140626	40141010	384	-42	MPLKIP	NM_138701	29.2829	no	no	M-phase specific PLK1 interacting protein
chr7	138468783	138469167	384	-55	TTC26	NM_001144920	0.000130524	yes	no	tetratricopeptide repeat domain 26
chr11	118471197	118471581	384	-2	H2AFX	NM_002105	38.6391	yes	yes	H2A histone family, member X
chr20	26137857	26138170	313	-144	MIR663AHG	NR_040095	0.0333671	yes	yes	MIR663A host gene (non-protein coding)
chr7	91601752	91602136	384	51	CYP51A1	NM_001146152	1.48747	yes	no	cytochrome P450, family 51, subfamily A, polypeptide 1
chr10	54669081	54669465	384	-467807	MBL2	NM_000242	0.009975	yes	yes	mannose-binding lectin (protein C) 2, soluble
chr1	159766586	159766970	384	6118	HSPA6	NM_002155	0.329236	no	no	heat shock 70kDa protein 6 (HSP70B')
chr3	187983691	187984075	384	-172	EIF4A2	NM_001967	56.7527	no	no	eukaryotic translation initiation factor 4A2
chr1	148125086	148125470	384	129	HIST2H2AC	NM_003517	4.05603	yes	no	histone cluster 2, H2ac
chrX	39824953	39825337	384	16518	BCOR	NM_017745	9.61676	yes	yes	BCL6 corepressor
chrX	21767435	21767819	384	50	MBTPS2	NM_015884	11.6643	yes	no	membrane-bound transcription factor peptidase, site 2
chrX	39898970	39899354	384	22364	BCOR	NM_001123383	0.000129958	yes	yes	BCL6 corepressor
chr1	161558054	161558438	384	-41	RGS5	NM_001254748	0.0971713	no	no	regulator of G-protein signaling 5
chr6	52968433	52968817	384	-488	GSTA4	NM_001512	2.16053	no	no	glutathione S-transferase alpha 4
chr9	113401770	113402154	384	-6	PTGR1	NM_001146109	4.89583	yes	yes	prostaglandin reductase 1
chr2	216587920	216588304	384	-1521	MREG	NM_018000	1.59775	yes	yes	melanoregulin
chr9	72938582	72938966	384	-12440	TRPM3	NM_001007471	8.33E-25	yes	yes	transient receptor potential cation channel, subfamily M, member 3
chr14	102908113	102908497	384	-13149	MARK3	NM_001128919	1.2647	yes	yes	MAP/microtubule affinity-regulating kinase 3
chr22	22737430	22737777	347	-162	CABIN1	NM_012295	6.66254	yes	yes	calcineurin binding protein 1
chr13	72534770	72535154	384	4031	KLF5	NM_001730	46.0319	yes	yes	Kruppel-like factor 5 (intestinal)
chr7	98475773	98475985	212	27155	LOC101927550	NR_110102	-	yes	yes	uncharacterized LOC101927550
chr12	107974550	107974934	384	-16	USP30	NM_032663	2.31702	no	no	ubiquitin specific peptidase 30
chr8	100005089	100005473	384	18694	STK3	NM_001256312	1.32E-06	yes	yes	serine/threonine kinase 3
chrX	24300017	24300401	384	9410	SUPT20HL1	NM_001136234	0	yes	yes	suppressor of Ty 20 homolog (S. cerevisiae)-like 1
chrX	39758431	39758815	384	83040	BCOR	NM_017745	9.61676	yes	yes	BCL6 corepressor
chr2	233058727	233059111	384	1894	ECEL1	NM_004826	33.6786	yes	no	endothelin converting enzyme-like 1
chr21	39607380	39607764	384	10	BRWD1	NM_001007246	1.6847	yes	no	bromodomain and WD repeat domain containing 1
chr14	66762190	66762574	384	-15383	MPP5	NM_001256550	5.07209	yes	yes	membrane protein, palmitoylated 5 (MAGUK p55 subfamily member 5)
chrY	10647660	10648044	384	289445	TTTY23	NR_001540	0	no	no	testis-specific transcript, Y-linked 23 (non-protein coding)
chr6	27829022	27829406	384	-8288	LOC100131289	NR_038929	0.0540982	yes	no	uncharacterized LOC100131289
chr2	85620174	85620558	384	754	MAT2A	NM_005911	113.641	yes	no	methionine adenosyltransferase II, alpha
chr1	63761390	63761774	384	-19	EFCAB7	NM_032437	2.46048	yes	no	EF-hand calcium binding domain 7
chr5	45018848	45019232	384	174256	MRPS30	NM_016640	54.2862	no	no	mitochondrial ribosomal protein S30
chr5	117077181	117077565	384	-16582	LOC102467224	NR_104997	-	yes	no	uncharacterized LOC102467224
chr7	6490184	6490568	384	-2	KDELR2	NM_006854	84.0391	yes	yes	KDEL (Lys-Asp-Glu-Leu) endoplasmic reticulum protein retention receptor 2
chr12	105275456	105275840	384	82	POLR3B	NM_018082	4.99783	yes	no	polymerase (RNA) III (DNA directed) polypeptide B
chr6	64341046	64341430	384	1362	PTP4A1	NM_003463	143.695	yes	no	protein tyrosine phosphatase type IVA, member 1
chr12	1159519	1159903	384	152536	ERC1	NM_001301248	-	yes	yes	ELKS/RAB6-interacting/CAST family member 1
chr1	143731753	143732137	384	19407	PDE4DIP	NM_001198832	0.535115	no	no	phosphodiesterase 4D interacting protein
chr7	96906928	96907312	384	-292087	TAC1	NM_003182	1.21E-07	no	no	tachykinin, precursor 1
chr1	61295148	61295434	286	-20243	NFIA	NM_001145511	0.0294951	yes	yes	nuclear factor I/A
chr18	75883593	75883977	384	-11549	RBFA	NM_001171967	0.450634	no	no	ribosome binding factor A (putative)
chr9	107163951	107164335	384	-85993	FSD1L	NM_001287191	-	yes	yes	fibronectin type III and SPRY domain containing 1-like
chr9	80618512	80618896	384	331697	LOC101927450	NR_109771	-	yes	yes	uncharacterized LOC101927450
chr6	41545730	41546114	384	-32238	LINC01276	NR_120347	-	yes	yes	long intergenic non-protein coding RNA 1276
chr12	123977670	123978054	384	11816	MIR5188	NR_049820	0	yes	no	microRNA 5188
chr15	35180662	35181046	384	-62	MEIS2	NM_002399	1.24434	yes	yes	Meis homeobox 2
chr11	73176389	73176773	384	16	MRPL48	NM_016055	49.6543	yes	no	mitochondrial ribosomal protein L48
chr5	169464288	169464667	379	-1018	FOXI1	NM_012188	0	yes	yes	forkhead box I1
chr1	147490620	147490718	98	-63085	LOC388692	NR_111933	-	no	no	uncharacterized LOC388692
chr2	3600393	3600777	384	-143	RPS7	NM_001011	1055.33	no	no	ribosomal protein S7
chr12	12311182	12311566	384	-296	LRP6	NM_002336	4.64071	yes	no	low density lipoprotein receptor-related protein 6
chr5	92950070	92950454	384	5463	NR2F1	NM_005654	41.5182	yes	yes	nuclear receptor subfamily 2, group F, member 1
chr2	55500386	55500770	384	-17	CCDC88A	NM_001254943	0.377183	yes	yes	coiled-coil domain containing 88A
chr1	113637319	113637703	384	-87113	LOC643441	NR_038846	0.0170802	yes	yes	uncharacterized LOC643441
chr17	8030781	8031165	384	-15	MIR4521	NR_039746	0	yes	yes	microRNA 4521
chr7	1576081	1576465	384	38	PSMG3-AS1	NR_021487	0.0923019	yes	no	PSMG3 antisense RNA 1 (head to head)
chr7	141084362	141084746	384	-36	SSBP1	NM_001256513	39.5478	yes	no	single-stranded DNA binding protein 1, mitochondrial

Chr	Start	End	Peak Size(bp)	Distance to TSS (bp)	Gene name	Nearest promoter ID	FPKM	ZDR(-0.08)	ZDR(-0.07)	Gene description
chr1	28847562	28847946	384	55	RNU11	NR_004407	15.3394	no	no	RNA, U11 small nuclear
chrX	13215158	13215542	384	-23342	GS1-600G8.3	NR_046087	0	yes	yes	unknown transcript
chr2	6902965	6903349	384	20244	CMPK2	NM_207315	2.05E-06	no	no	cytidine monophosphate (UMP-CMP) kinase 2, mitochondrial
chr12	42181361	42181745	384	50438	ADAMTS20	NM_025003	0	yes	yes	ADAM metallopeptidase with thrombospondin type 1 motif, 20
chr5	29493028	29493412	384	-61380	LOC101929681	NR_104628	-	yes	yes	uncharacterized LOC101929681
chr5	36451397	36451781	384	-113821	RANBP3L	NM_145000	0	yes	yes	RAN binding protein 3-like
chr1	54478181	54478565	384	3669	SSBP3-AS1	NR_103541	-	yes	yes	SSBP3 antisense RNA 1
chr5	71835476	71835860	384	3337	ZNF366	NM_152625	0.0130863	yes	yes	zinc finger protein 366
chr10	101055509	101055893	384	-23145	CNNM1	NM_020348	2.88553	yes	yes	cyclin and CBS domain divalent metal cation transport mediator 1
chr12	119122917	119123301	384	288	RPLP0	NM_001002	1456.12	yes	no	ribosomal protein, large, P0
chrX	47226788	47227172	384	309	ZNF41	NM_007130	1.25003	yes	yes	zinc finger protein 41
chr16	48954861	48955245	384	5293	BRD7	NM_013263	22.2527	yes	yes	bromodomain containing 7
chr17	11376339	11376723	384	-65942	DNAH9	NM_001372	0.00316562	yes	yes	dynein, axonemal, heavy chain 9
chr1	159848996	159849380	384	6715	HSPA7	NR_024151	0.0733645	no	no	heat shock 70kDa protein 7 (HSP70B)
chr1	222760399	222760783	384	-71967	WDR26	NM_001115113	6.74189	yes	yes	WD repeat domain 26
chr1	178467124	178467508	384	1260	LHX4	NM_033343	1.22399	yes	yes	LIM homeobox 4
chr1	159635944	159636328	384	-31839	C1orf192	NM_001013625	6.04479	no	no	chromosome 1 open reading frame 192
chr10	99428361	99428745	384	8452	AVPI1	NM_021732	100.03	yes	yes	arginine vasopressin-induced 1
chrX_random	1541885	1542269	384	-129947	CRLF2	NR_110830	-	yes	no	cytokine receptor-like factor 2
chr1	65305986	65306370	384	-9399	MIR101-1	NR_029516	0	yes	yes	microRNA 101-1
chr15	88238079	88238463	384	350	AP3S2	NR_023361	1.20825	yes	no	adaptor-related protein complex 3, sigma 2 subunit
chr1	88650441	88650825	384	-271877	PKN2	NM_006256	9.23972	yes	yes	protein kinase N2
chr2	42891044	42891428	384	-17981	HAAO	NM_012205	0.124659	yes	no	3-hydroxyanthranilate 3,4-dioxygenase
chr10	106337096	106337480	384	-53561	SORCS3	NM_014978	0.0181247	yes	yes	sortilin-related VPS10 domain containing receptor 3
chr19	1063488	1063872	384	8031	GPX4	NM_001039848	11.654	yes	yes	glutathione peroxidase 4
chr8_random	325865	326249	384	-73330	SCRT1	NM_031309	0.0200514	no	no	scratch family zinc finger 1
chr19	1201688	1202072	384	2328	MIDN	NM_177401	16.6974	yes	no	midnolin
chr17	60549697	60550081	384	-14029	RGS9	NM_001165933	0.0231238	yes	no	regulator of G-protein signaling 9
chr21	43661200	43661584	384	10038	SIK1	NM_173354	105.685	yes	no	salt-inducible kinase 1
chrX	70204852	70205236	384	-21	SNX12	NM_013346	91.8538	yes	yes	sorting nexin 12
chr17	44624717	44625101	384	16833	GNGT2	NM_031498	1.65E-05	no	no	guanine nucleotide binding protein (G protein), gamma transducing activity polypeptide 2
chr5	36912972	36913356	384	546	NIPBL	NM_133433	13.9417	no	no	Nipped-B homolog (Drosophila)
chr11	8848460	8848844	384	40422	ST5	NM_005418	0.246697	yes	yes	suppression of tumorigenicity 5
chr1	148726130	148726514	384	-142	TARS2	NM_025150	19.0135	yes	no	threonyl-tRNA synthetase 2, mitochondrial (putative)
chr8	125808332	125808716	384	1405	MTSS1	NM_001282971	-	yes	yes	metastasis suppressor 1
chr14	60271047	60271431	384	27	MNAT1	NM_002431	10.2768	yes	no	MNAT CDK-activating kinase assembly factor 1
chr7	47985549	47985933	384	6	HUS1	NM_004507	3.50169	yes	no	HUS1 checkpoint homolog (S. pombe)
chr6	25389131	25389515	384	1688	LRRC16A	NM_001173977	3.54123	yes	yes	leucine rich repeat containing 16A
chr7	135307309	135307693	384	5243	MTPN	NM_145808	-	yes	yes	myotrophin
chr7	148053037	148053421	384	26363	CUL1	NM_003592	24.3097	yes	yes	cullin 1
chr6	166321235	166321619	384	90	LINC00473	NR_026861	40.9784	yes	yes	long intergenic non-protein coding RNA 473
chr15	57518416	57518800	384	944	FAM81A	NM_152450	3.0338	yes	yes	family with sequence similarity 81, member A
chr2	46696505	46696889	384	1058	PIGF	NM_002643	7.56921	yes	yes	phosphatidylinositol glycan anchor biosynthesis, class F
chr8	140781842	140782038	196	2541	KCNK9	NR_104210	-	yes	yes	potassium channel, subfamily K, member 9
chr5	173058432	173058816	384	47194	LINC01484	NR_108027	-	yes	yes	long intergenic non-protein coding RNA 1484
chr10	95452221	95452605	384	-94	FRA10AC1	NM_145246	2.67654	yes	no	fragile site, folic acid type, rare, fra(10)(q23.3) or fra(10)(q24.2) candidate 1
chr6	26629271	26629655	384	-450	HCG11	NR_026790	5.98881	yes	no	HLA complex group 11 (non-protein coding)
chr22	48741521	48741905	384	-805	MIR6821	NR_106879	-	yes	yes	microRNA 6821
chr21	32906514	32906898	384	78	C21orf59	NR_036552	2.51549	no	no	chromosome 21 open reading frame 59
chr12	47740604	47740988	384	-5422	KMT2D	NM_003482	10.7047	yes	no	lysine (K)-specific methyltransferase 2D
chr1	59023393	59023777	384	174	LINC01135	NR_034015	0	yes	no	long intergenic non-protein coding RNA 1135
chr12	119215084	119215468	384	-9231	SIRT4	NM_012240	0.33467	yes	no	sirtuin 4
chr12	47544289	47544673	384	1439	RND1	NM_014470	0.230133	yes	yes	Rho family GTPase 1
chr2	112634619	112635003	384	22378	FBLN7	NM_153214	0.983214	yes	yes	fibulin 7
chr12	102883801	102884185	384	270	TDG	NM_003211	8.50306	yes	yes	thymine-DNA glycosylase
chr10	3817567	3817951	384	-286	KLF6	NM_001160124	0.219137	yes	yes	Kruppel-like factor 6
chr1	148124266	148124650	384	398	HIST2H2BE	NM_003528	2.85087	yes	no	histone cluster 2, H2be
chr3	170972561	170972945	384	-794	MYNN	NM_018657	0.584903	no	no	myoneurin
chr4	141294896	141295280	384	-405	MAML3	NM_018717	1.49592	yes	yes	mastermind-like 3 (Drosophila)
chr17	35390531	35390915	384	176	PSMD3	NM_002809	115.748	yes	no	proteasome (prosome, macropain) 26S subunit, non-ATPase, 3
chrX	136788003	136788387	384	312183	ZIC3	NM_003413	0	yes	yes	Zic family member 3
chr12	45051890	45052274	384	830	SLC38A2	NM_018976	129.684	yes	yes	solute carrier family 38, member 2
chr4	103642425	103642809	384	1099	NFKB1	NM_001165412	3.35969	yes	yes	nuclear factor of kappa light polypeptide gene enhancer in B-cells 1
chr7	138238972	138239356	384	77440	KIAA1549	NM_020910	1.07837	yes	yes	KIAA1549
chr16	22217194	22217578	384	1189	POLR3E	NM_001258035	1.9765	no	no	polymerase (RNA) III (DNA directed) polypeptide E (80kD)
chr14	54103023	54103407	384	-865	SAMD4A	NM_001161576	0.744408	yes	yes	sterile alpha motif domain containing 4A
chr20	26136785	26137220	435	-88	MIR663A	NR_030386	2025.68	yes	no	microRNA 663a
chr1	204852261	204852645	384	74	EIF2D	NM_006893	35.6064	yes	no	eukaryotic translation initiation factor 2D
chr1	145022683	145023067	384	52	RNVU1-8	NR_121645	-	yes	no	RNA, variant U1 small nuclear 8
chr5	149935463	149935568	105	-25320	SYNPO	NM_001166208	0.000364454	yes	yes	synaptopodin
chr21	43670780	43670953	173	564	SIK1	NM_173354	105.685	yes	no	salt-inducible kinase 1
chr14	73292565	73292949	384	-2446	MIR4505	NR_039727	0	yes	yes	microRNA 4505
chr16	79395461	79395845	384	23	CDYL2	NM_152342	7.57569	yes	yes	chromodomain protein, Y-like 2
chr1	148249162	148249546	384	-44	OTUD7B	NM_020205	8.98092	no	no	OTU deubiquitinase 7B
chr3	151803837	151804221	384	273	SELT	NM_016275	10.8076	yes	no	selenoprotein T
chr1	224965545	224965929	384	27762	ITPKB	NM_002221	4.07654	yes	yes	inositol-trisphosphate 3-kinase B
chr19_random	1	371	371	-164408	KIR2DS2	NM_012312	-	no	no	ke receptor, two domains, short cytoplasmic tail, 2

Supplementary Table S4. List of genes nearest to 10 ZFSs including predicted ZDRs.

Gene name	ZDR location	ZDR length	ZDR Sequence
**Alternating (Pu)(Py) group**			
ANKRD11	Promoter	60	ACACACACACACACACGCGCGCGCGCGCGCGCGCACACACATACACCACACACACAGC
ROR1	Promoter	48	GGGTGTGCACGCGCGCGCGCGCGCGTGTGTGTGTGCGTGTTACAAACA
SNX12	Promoter	45	GCGCGCACGCGCGCCCACGCACGCACGCGCACGCCGCACGGGCCTG
SRSF6	Promoter	46	GCGCGCGCGCGCGCCATTGTGTGGCTGGACTCGGCCGCCCCTGTGG
TFAP2A	Promoter	20	GTGGGTGCGTGCGTGTTCCT
**Non-alternating (Pu)(Py) group**			
**Gene name**	**ZDR location**	**ZDR length**	**ZDR Sequence**
SIK1	Gene body	28	GCGCGCACCTGCGGGGCCGCACAGAGCT
HIST2H2AC	Promoter	32	GGGCCGGCGCGCCCGTCTACATGGCGGCGGTC
PLK2	Promoter	12	GCGCGGTCACAC
STX16	Promoter	16	GCACTCCCGTGCCCCG
PIM3	Promoter	14	GCGGCCGCGCGCCC
**Negative control**			
**Gene name**	**ZDR location**	**ZDR length**	**Sequence**
–	–	28	GCCGGCCGCCGGCCGCCGGCCGCCGGCC

Gene are classified into two groups according to the number of alternating purine/pyrimidine dinucleotide in predicted ZDRs; an alternating (Pu)(Py) (≥6 dinucleotides) and a non-alternating (Pu)(Py) group. Negative control is a arbitrary sequence which has no predicted ZDR.

Supplementary Table S5. The number of RNA polymerase II peaks overlappingwith ZFSs.

	**Pol II-1**	**Pol II-2**	**Pol II-3**
total peaks	25,921	34,160	26,496
overlap with ZFSs	200	210	200
ZFSs[1]		231	

Three RNA polymerase II ChIP-Seq datasets which were generated from different groups were marked as Pol II-1, Pol II-2, and Pol II-3. [1] ZFSs were counted when they overlapped with RNA polymerase II peaks found in all three RNA polymerase II ChIP-Seq datasets.
